# Variation of floret fertility in hexaploid wheat revealed by tiller removal

**DOI:** 10.1093/jxb/erv303

**Published:** 2015-07-08

**Authors:** Zifeng Guo, Thorsten Schnurbusch

**Affiliations:** ^1^HEISENBERG Research Group Plant Architecture, Leibniz Institute of Plant Genetics and Crop Plant Research (IPK), Corrensstr. 3, 06466 Stadt Seeland, OT Gatersleben, Germany

**Keywords:** Fertile floret, final grain, floral degradation, maximum number of floret primordia.

## Abstract

Grain number per spike, which is greatly influenced by floret fertility, is an important trait of wheat (*Triticum aestivum* L.) yield. Maximum floret primordia, fertile floret, and final grain number per spikelet are three crucial factors of floret fertility. Floral degradation plays a critical role in determining these three floret fertility-related traits. Twelve hexaploid spring wheat genotypes were selected to investigate the influence of detillering on floral degradation and floret fertility-related traits in the field and greenhouse. Notably, the green anther stage was found to consistently have the maximum floret primordia number. Visible floral degradation, however, was observed to occur at several floral developmental stages, specifically from green anther stage to anthesis. Detillering was able to delay floral degradation in most cases and was evidently highly associated with increased maximum floret primordia, fertile floret, and final grain number per spikelet, with only a few exceptions. Thermal time required for each floral developmental stage was overall not influenced by detillering. These data hereby reveal a predominant spikelet fertility pattern along the spike in which the number of fertile florets per spikelet at anthesis becomes developmentally confined.

## Highlight

In hexaploid wheat, floret primordia number reaches maximum at green anther stage; visible floral degradation may occur at different stages and can be delayed by detillering.

## Introduction

Grass spikelets produce reproductive structures called florets; the determination of floret number per spikelet is a primary factor of spikelet architecture in the grass family. The indeterminate nature of wheat (*Triticum aestivum* L.) spikelets may enable more than eight florets to be formed within one spikelet, which is in contrast with other cereals that have a fixed number (*Oryza sativa* and *Zea mays*) (Skibbe *et al.*, 2008; Yoshida and Nagato, 2011) or effectively are fixed to one floret (*Hordeum vulgare*, barley) (Bonnett, 1967). Final grain number per spikelet (grain setting) at physiological maturity is one of the most decisive factors for final grain yield in wheat (Reynolds *et al.*, 2009; Fischer, 2011; Pedro *et al.*, 2012). However, before the final grain number is set at physiological maturity, floral structures undergo a sophisticated development and abortion process. After reaching the maximum number of floret primordia, representing wheat’s yield potential, a floral degradation process is initiated determining the fertile florets at anthesis. Following this pre-anthesis floral degradation, another one to three florets are usually lost during post-anthesis events until final grain number is reached at physiological maturity (PM). Maximum floret primordia number, fertile floret number, and final grain number per spikelet are three crucial points during this floral developmental process. Previous studies monitored the floral degradation process according to the Waddington scale (Waddington *et al.*, 1983). For example, Craufurd and Cartwright (1989) reported that floret death began when the floret 1 (F1, the first floret from the spikelet base) in the central spikelet reached a stage of Waddington score 8 (W8). Bancal (2009) observed the onset of floret abortion during a period ranging between W7 (styles elongating) to W8. González et al. (2011) reported the initiation of floret death when F1 ranged from W8 to W9. As far as it could be ascertained, the only work to determine the onset of floral degradation following the Kirby scale was conducted by Kirby and Appleyard. They showed that floret primordia reach a maximum at the white anther stage (Kirby and Appleyard, 1987). Although previous studies have shown that exogenous and endogenous factors can affect final grain yield in wheat (McGrath *et al.*, 1997; Richards *et al.*, 2002; Sadras and Denison, 2009; Richards *et al.*, 2010; Potgieter *et al.*, 2013; Sadras and Richards, 2014), more work is needed to enrich our knowledge of how these factors influence floral development and abortion processes, especially the initiation of floral degradation, which will determine final grain number.

Tillering is a critical factor for wheat yield. It is regulated genotypically, but is also affected by the environment (Pinthus and Meiri, 1979; Spielmeyer and Richards, 2004; Duggan *et al.*, 2005; Kuraparthy *et al.*, 2007; Fujita *et al.*, 2010; Mitchell *et al.*, 2012; Dreccer *et al.*, 2013). Tillering is closely associated with wheat yield because of its involvement in grain number and grain weight determination (Mohamed and Marshall, 1979; Kemp and Whingwiri, 1980; Borras-Gelonch *et al.*, 2012). An excessive tiller number in wheat can result in yield reductions because some tillers compete for assimilates with the main shoot but abort before reaching physiological maturity and thus do not contribute to the final grain yield (Ishag and Taha, 1974; Thorne and Wood, 1987; Davidson and Chevalier, 1990). Low tiller numbers can also lead to yield loss because of reduced spike and grain number. Although grain weight can increase in this case, it is not enough to compensate for the loss caused by tiller reduction.

Previously, a wheat mutant with strongly reduced tillering was identified. This so-called *tiller inhibition* (*tin*) mutant had thicker stems, a higher kernel number per unit stem weight, a lower leaf to stem weight ratio at maturity, and larger spikes with more and larger grains (Atsmon and Jacobs, 1977; Richards, 1988). Research was conducted to determine the physiological characteristics of *tin* mutants across environments and treatments. The growth of *tin* mutants was found to be stunted after long photoperiods and at low temperatures (Atsmon *et al.*, 1986; Duggan *et al.*, 2002). Their internodes are solid rather than hollow, suggesting that tiller bud growth is arrested owing to sucrose transfer from the bud to support internode elongation (Kebrom *et al.*, 2012; Kebrom and Richards, 2013; Kebrom *et al.*, 2013; Kebrom and Mullet, 2014). Interestingly, the effects of *tin* on grain yield were found to vary with environment and genetic background. Grain yield was unchanged in some *tin* lines, but reduced in others (Mitchell *et al.*, 2012). Although nitrogen increased spike numbers in *tin* lines, these numbers were still around 20% lower than those of free-tillering cultivars (Duggan *et al.*, 2005). The higher grain weight of *tin* mutant lines under stress conditions was related to more biomass accumulation at anthesis and increased levels of water-soluble carbohydrates in the stem, which ensures more assimilate for later diversion to grain filling (Mitchell *et al.*, 2012).

In addition to studies on reduced-tillering (*tin*) wheat lines, a number of works have been conducted to investigate tiller removal. Results of detillering experiments in wheat and barley have shown considerable increase in grain yield (including grain number and weight), main stem dry weight, and total biomass. This overall response after tiller removal indicates that tillers compete with the main shoot for resources, which significantly restricts improvements in the potential biomass and yield (Mohamed and Marshall, 1979; Kemp and Whingwiri, 1980; Elalaoui *et al.*, 1988; Gu and Marshall, 1988; Kirby *et al.*, 1994). Although the influence of tiller removal on grain yield-related traits has been shown, there is a paucity of information regarding its effects on the maximum number of floret primordia and fertile floret number in wheat. Furthermore, work related to the effects of tiller removal on floral degradation is not well documented.

Field growing conditions usually induce a complex environmental response in plants due to steadily fluctuating growing conditions, whereas the greenhouse environment is comparably stable. Thus, it is necessary to determine the effects of detillering on the maximum number of floret primordia, fertile floret number, final grain number, and floral degradation under field and greenhouse growth conditions. To this end, floret primordia number was measured at seven floral developmental stages to determine the timing of maximum floret primordia number. Comparisons between control and tiller removal experiments were conducted to show the effects of detillering on the timing of floral degradation, maximum floret primordia number, fertile floret number, and final grain number.

## Materials and methods

### Plant materials and growth conditions

Experiments were carried out at the Leibniz Institute of Plant Genetics and Crop Plant Research, Gatersleben, Germany (51° 49′ 23″ N, 11° 17′ 13″ E, altitude 112 m) during the 2014 growing season under greenhouse and field conditions. Twelve German hexaploid spring wheat cultivars were selected according to their years of release (Table 1). Control and tiller removal experiments were conducted in the field and greenhouse simultaneously. Tillers were removed two to three times per week. Eighty plants per cultivar (forty plants for control and forty plants for tiller removal) were planted under field and greenhouse conditions.

**Table 1.** German wheat cultivars studied and their years of release to the market.

Running number	Cultivar name	Year of release
1	Adlungs Alemannen	1931
2	NOS Nordgau	1933
3	Peragis Garant	1946
4	Heines Peko	1947
5	Hohenheimer Franken II	1951
6	Probat	1953
7	Breustedts Lera	1959
8	Arin	1962
9	Kolibri	1966
10	Ralle	1980
11	Nandu	1988
12	Fasan	1997

For both greenhouse and field experiments, seeds were sown in 96-well trays on the same date (11 February 2014) and germinated under greenhouse conditions (photoperiod, 16h/8h, light/dark; temperature, 20°C/16°C, light/dark) for 14 days. Seedlings at the two- to three-leaf stage were transferred to 4°C to vernalize for 63 days. Vernalized seedlings were transferred to a hardening stage (photoperiod, 12h/12h, light/dark; temperature, 15°C) for 7 days to gradually acclimatize. Finally, half of the plants were transplanted into 0.5L pots (one plant per pot; 9×9 × 9cm) under greenhouse conditions (photoperiod, 16h/8h, light/dark; temperature, 20°C/6°C, light/dark) (Supplementary Fig. S1a). Supplemental light (~250 μmol m^−2^ s^−1^ photosynthetically active radiation) was supplied with low-intensity incandescent light and plants were irrigated when required. The other half of the plants were directly planted into a field with silty loam soil (20 plants per 2 m-long row with 20cm between rows) (Supplementary Fig. S1b). All plants were manually irrigated on requirement. The temperature and global solar radiation in the 2014 field growing season are presented in Supplementary Table S1.

### Phenotypic staging and measurements

To study detillering on floral degradation, maximum number of floret primordia, fertile floret number, and final grain number, seven floral developmental stages were selected: terminal spikelet (TS) stage (completion of spikelet initiation; Kirby and Appleyard, 1987); white anther (WA) stage (lemmas of F1 and F2 completely enclose stamens and other structures; Kirby and Appleyard, 1987); green anther (GA) stage (glumes cover all but the tips of florets; Kirby and Appleyard, 1987); yellow anther (YA) stage (glumes are fully formed and the lemmas of the first three florets are visible; Kirby and Appleyard, 1987); tipping (TP) stage (Z49, first awns visible; Zadoks *et al.*, 1974); heading (HD) stage (Z55, 50% of spikes visible; Zadoks *et al.*, 1974); and anthesis (AN) stage (Z65, 50% of spikes with anthers; Zadoks *et al.*, 1974). The corresponding Waddington stages of F1 at the seven floral developmental stages are shown in Supplementary Table S2 to be better able to compare between this study and others.

In order to detect the TS, WA, GA, and YA stages, every cultivar was examined every two days under a stereomicroscope (Stemi 2000-c, Carl Zeiss Micro Imaging GmbH, Gottingen, Germany). For the TP, HD, and AN stages, the day of onset was recorded as the point at which 50% of plants reached that particular stage. Thermal time was used to identify the duration of each stage and was calculated as the sum of the daily average temperature [(Tmax + Tmin) / 2] (a base temperature was assumed as 0°C).

**Fig. 1.**
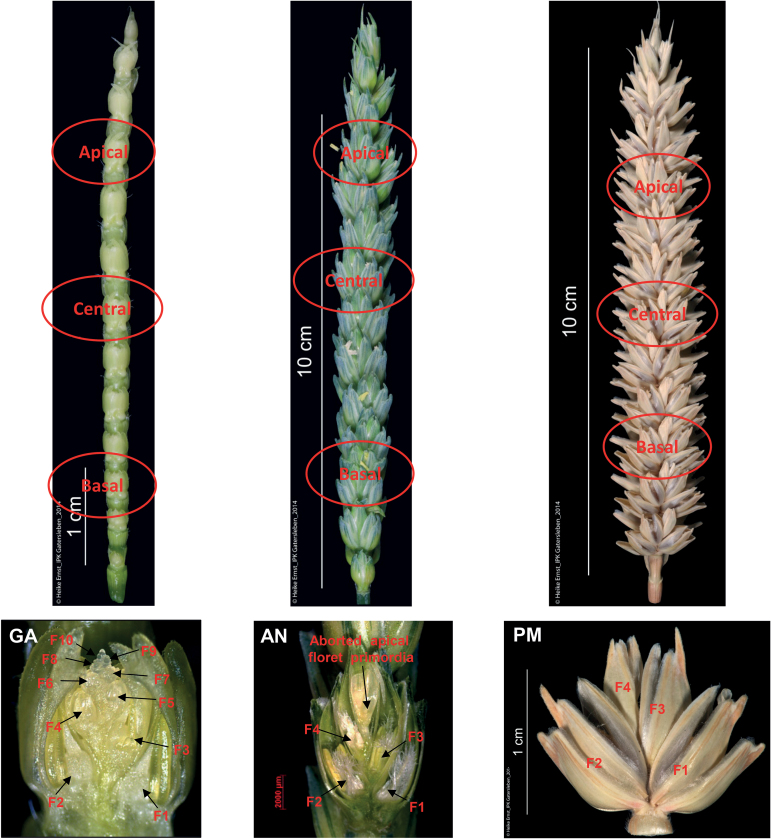
Apical, central, and basal spikelets for measurement of maximum number of floret primordia (GA), fertile floret number (AN), and final grain number (PM) per spikelet.

**Fig. 2.**
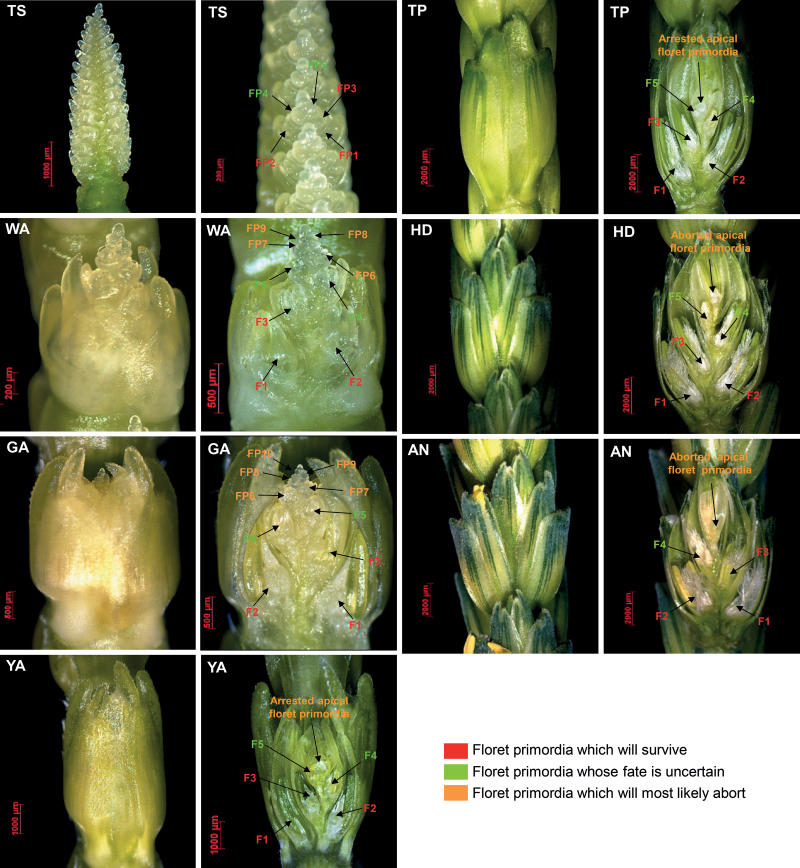
Details of floral development and abortion processes at seven developmental stages in wheat following the staging of Kirby and Appleyard (1987). The left panels are spikelets at the seven floral developmental stages, and the right panels are the longitudinal section of corresponding spikelets.

**Fig. 3.**
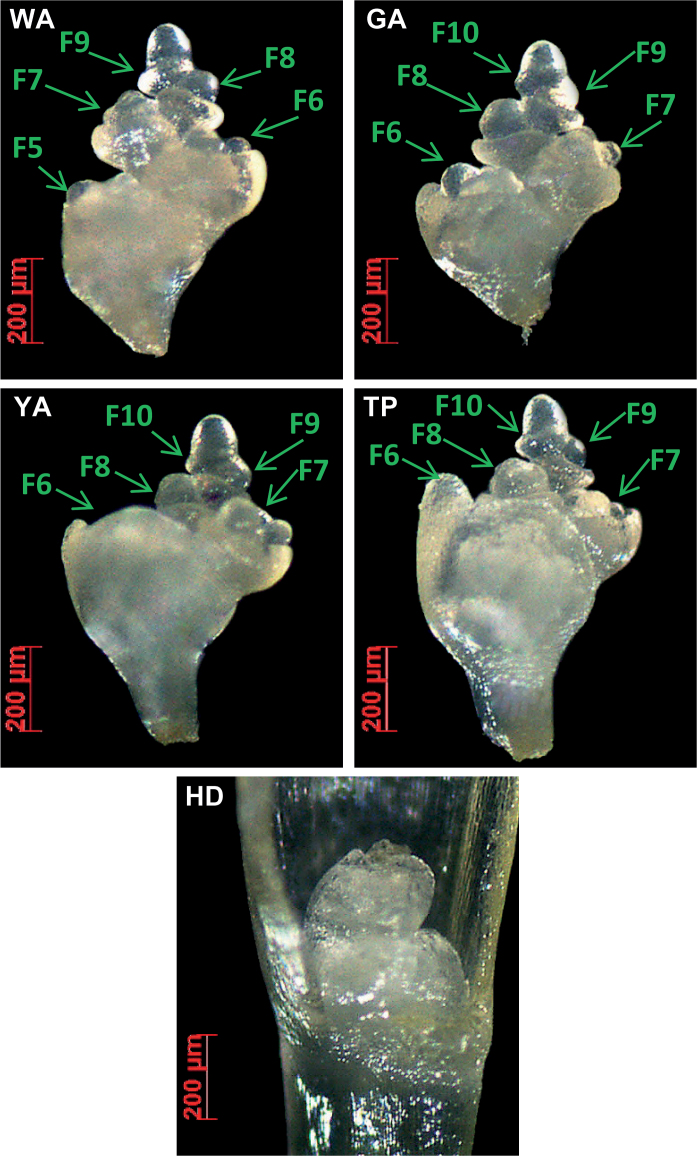
Floret primordia growth, arrest and visible degradation at WA, GA, YA, TP, and HD stages. At WA stage, the first four florets at the bottom were removed; at GA, YA, TP, and HD stages, the first five florets at the bottom were removed. Exemplified is the process of floret primordia arrest (GA to YA) and visible degradation (TP to HD); due to genotypic variation, the visual degradation can vary from GA, YA, TP or HD.

**Fig. 4.**
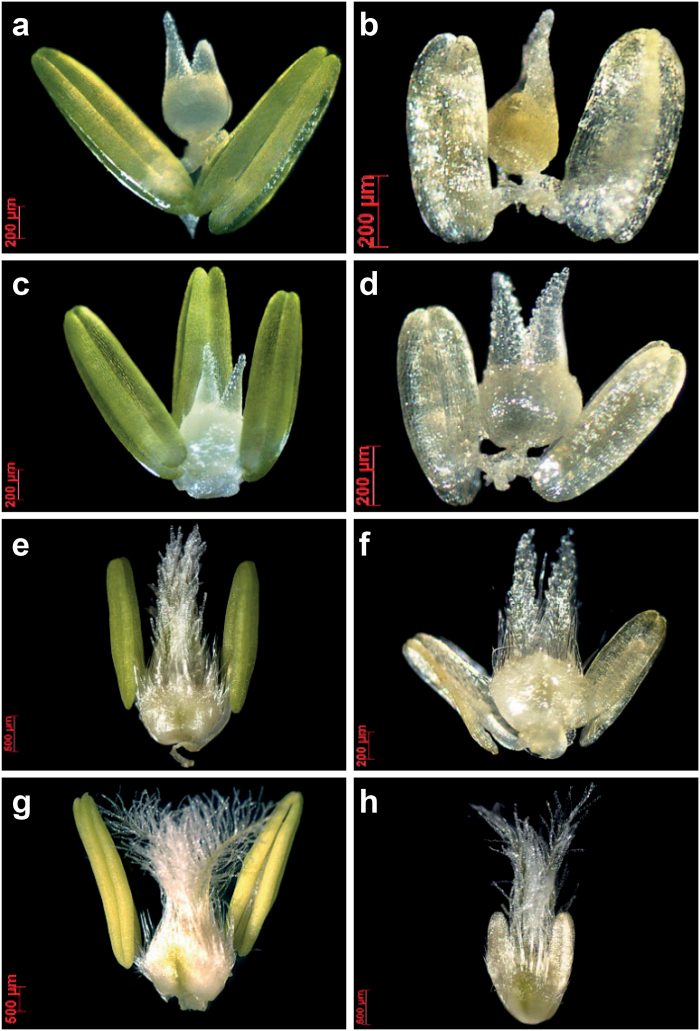
Fertile (left, a, c, e, g) and aborted florets (right, b, d, f, h) at the same floral developmental stages. Please note that these fertile and aborted florets can be found at different floret positions within the same spikelets or at different floral developmental stages at the same floret positions in different spikelets.

During each stage, main shoots of three plants for each cultivar were randomly selected to measure floret primordia and fertile floret number per spikelet, while main shoots of six plants were used for determining final grain number per spikelet. Floret primordia, fertile floret, and final grain number per spikelet were measured in spikelets at three positions: apical (the third spikelet from the top of spike), central (the spikelet in the centre of the spike), and basal positions (the third spikelet from the bottom of the spike) (Fig. 1) at different floral developmental stages (Fig. 2). It should be noted that apical and basal spikelets of the spike in this experiment were high-middle and low-middle positions of the spike and did not include the extreme apical and basal spikelets at the top and bottom of the spike. The number of floret primordia per spikelet at the apical parts of the spikelets was not visible from YA to HD stage compared with basal parts of spikelets (Fig. 2), so the apical part was separated out to display apical floret primordia number (Fig. 3). Owing to genotypic variation in floral development, visible floral degradation (as seen in Fig. 3 from TP to HD) can also occur at other stages between GA to AN. To determine the fertile floret number per spikelet at AN, it was necessary to distinguish between living and aborted florets. The living and aborted florets at the same developmental stages are shown in Fig. 4; the anthers in the aborted florets are always small and dry, and the stigmatic hairs are not well-developed. Leaf area of the main stem at GA and AN stage was measured immediately after dissection of fresh leaf material using an area meter (LI-3100, LI-COR Ltd., Nebraska, USA). The main stem, tillers, and spike on the main shoot at GA and AN stage were dried in two cellophane bags at 60°C for 3–5 days for dry weight measurement and they were measured directly after harvesting for physiological maturity (PM) stage. Stem dry weight refers to the dry weight of shoots without spikes. In this experiment, floret and grain number, leaf area, and spike dry weight were only measured on the main shoot.

## Results

### Influence of detillering on visible floral degradation

The timing of visible floral primordia degradations are shown in grey frames in Tables 2, 3, 4, and 5 and are indicated from TP to HD stage in Fig. 3. As shown in Table 2, visible floral degradation in the four exemplified genotypes occurred from GA to YA in ‘NOS Nordgau’, YA to TP in ‘Adlungs Alemannen’, TP to HD in ‘Peragis Garant’, and HD to AN in ‘Nandu’, indicating that visible floral degradation can occur at all floral developmental stages between GA and AN. Tables 3 and 5 show that visible floral degradation in three genotypes (NOS Nordgau, Adlungs Alemannen, and Peragis Garant) grown in the field and in the greenhouse was delayed by tiller removal relative to their respective environmental controls (Tables 2 and 4). Additionally, Table 4 (grey frames) shows that visible floral degradation without tiller removal was delayed by greenhouse growth conditions compared with control plants in the field (Table 2). A delay in floral degradation was not found in Nandu plants because it occurred from HD to AN, which is the last stage for fertile florets. Thus, floral degradation can be delayed by both tiller removal and greenhouse growth conditions (Table 2 and 3; Supplementary Tables S3 and S4). It should also be noted that GA stage contained the maximum floret primordia number across the two environments (field and greenhouse), the two treatments (control and tiller removal), and all 12 tested genotypes (Supplementary Tables S5, S6, S7, and S8). Generally, the difference between floret primordia and fertile floret number in spikelet positions of the same developmental stage was below two; in most cases, it was one or zero (the standard deviation was below two). Moreover, visible floral degradation in apical, central, and basal spikelets occurred at the same stage within a genotype. However, in a few cases, the standard deviations of floret primordia number became very large, indicating large differences between plants within a genotype (Supplementary Tables S5, S6, S7, and S8). The explanation is that the floral degradation had already occurred in one selected plant, suggesting almost half of floret primordia lost, but not in the other two selected plants. In extreme cases, the three selected positions (apical, central, and basal) within one spike also displayed different degradation situations: for example, in the ‘Ralle’ cultivar, degradation occurred during HD in the apical and middle spikelets, but not in the basal spikelets (Supplementary Table S6).

**Table 2.** Living floret (primordia) number per spikelet in apical, central and basal spikelets at seven floral developmental stages and grain number per spikelet at physiological maturity (PM) in four selected, free-tillering genotypes (control) grown in the field.

**Cultivars**	**Position**	**TS**	**WA**	**GA**	**YA**	**TP**	**HD**	**AN**	**PM**
NOS Nordgau	Apical	2.00±0.00	8.67±0.58	**10.00±0.00**	4.00±0.00	4.00±0.00	4.00±0.00	3.00±0.00	2.83±0.75
	Central	3.67±0.58	9.00±0.00	**10.33±0.58**	5.67±0.58	5.00±0.00	5.00±0.00	4.33±0.58	3.67±0.82
	Basal	3.00±0.00	9.67±0.58	**10.33±0.58**	5.67±0.58	5.00±0.00	5.00±0.00	4.33±0.58	3.67±0.52
Adlung’s Alemannen	Apical	2.33±0.58	9.00±1.00	**9.67±0.58**	9.67±0.15	4.33±0.58	4.33±0.58	3.00±0.00	2.50±0.55
	Central	3.33±0.58	10.00±0.58	**10.67±0.58**	10.00±0.00	5.33±0.58	5.33±0.58	4.33±0.58	3.67±0.82
	Basal	3.33±0.58	9.33±0.58	**10.33±0.58**	11.00±0.00	5.33±0.58	5.00±1.00	4.33±0.58	3.50±0.55
Peragis Garant	Apical	4.00±0.00	8.67±1.15	**10.00±0.00**	9.33±1.15	9.67±1.15	4.00±0.00	3.67±0.58	2.67±0.52
	Central	5.33±0.58	10.33±0.58	**11.00±1.00**	11.00±1.00	10.67±1.15	5.67±0.58	5.00±1.00	3.50±0.84
	Basal	5.33±0.58	10.33±0.58	**11.33±0.58**	10.67±0.58	11.67±0.58	6.00±0.00	5.00±1.00	3.00±1.26
Nandu	Apical	3.33±0.58	9.00±0.00	**10.67±0.58**	10.33±0.58	10.00±0.00	10.00±0.00	3.33±0.58	2.50±0.55
	Central	5.33±0.58	10.00±0.00	**11.67±0.58**	11.33±0.58	11.00±0.00	11.00±0.00	4.33±0.58	3.67±0.52
	Basal	5.00±1.00	10.00±0.00	**11.67±0.58**	12.00±0.00	11.67±0.58	12.00±1.00	4.33±0.58	3.33±1.03

Data are presented as the mean ± SD, n=6 for PM stage, and n=3 for the remaining stages. The bold text suggests maximum floret primordia number stage is GA stage, and the grey boxes indicate the time windows of floral degradation occrued.

**Table 3.** Living floret (primordia) number per spikelet in apical, central and basal spikelets at seven floral developmental stages and grain number per spikelet at physiological maturity (PM) in the four selected, detillered genotypes (tiller removal) grown in the field.

**Cultivars**	**Position**	**TS**	**WA**	**GA**	**YA**	**TP**	**HD**	**AN**	**PM**
NOS Nordgau	Apical	2.00±0.00	8.33±0.58	**10.67±0.58**	10.33±0.58	9.67±0.58	5.67±0.58	3.67±0.58	3.00±1.55
	Central	3.33±0.58	9.00±1.00	**10.67±0.58**	11.00±0.00	11.00±1.00	6.33±0.58	6.00±0.00	4.50±0.84
	Basal	3.33±0.58	9.67±0.58	**10.67±0.58**	10.67±0.58	11.00±0.00	7.33±2.31	6.00±0.00	4.50±0.84
Adlung’s Alemannen	Apical	3.50±0.71	8.67±0.58	**10.67±0.58**	10.67±0.58	10.33±0.58	5.67±0.58	5.00±1.00	2.33±1.75
	Central	4.50±0.71	9.67±0.58	**10.67±0.58**	11.33±0.58	11.67±0.58	6.00±1.00	6.00±0.00	4.67±0.52
	Basal	4.00±0.00	9.67±0.58	**11.00±0.00**	11.33±0.58	12.00±0.00	5.67±0.58	6.00±0.00	4.33±1.21
Peragis Garant	Apical	4.00±1.73	9.67±0.58	**10.67±0.58**	10.67±0.58	10.67±0.58	9.00±3.46	3.33±0.58	2.50±1.05
	Central	5.33±0.58	10.33±0.58	**11.67±0.58**	12.00±0.00	11.33±0.58	10.00±3.46	5.00±0.00	3.33±1.03
	Basal	6.67±0.58	9.67±0.58	**11.67±0.58**	12.00±0.00	12.33±0.58	10.00±3.46	5.00±1.00	3.33±1.03
Nandu	Apical	3.67±1.15	8.00±0.00	**10.67±0.58**	11.00±0.00	11.00±0.00	11.00±1.00	4.67±0.58	3.29±0.76
	Central	5.67±0.58	9.00±0.00	**11.00±0.00**	12.33±0.58	12.00±0.00	12.33±0.58	6.67±0.58	4.00±0.82
	Basal	6.00±1.00	10.00±0.00	**11.33±0.58**	11.67±0.58	12.33±0.58	13.00±0.00	6.67±0.58	3.43±1.13

Data are presented as the mean ±SD, n=6 for PM stage, and n=3 for the remaining stages. The bold text suggests that maximum floret primordia number stage occurred during GA stage, while grey boxes indicate the time windows when visible floral degradation occurred.

**Table 4.** Stages of visible floral degradation in 12 spring wheat cultivars grown in the field (control and tiller removal).

Cultivars	Control + Field				Detillering + Field			
	GA-YA	YA-TP	TP-HD	HD-AN	GA-YA	YA-TP	TP-HD	HD-AN
1- Adlung’s Alemannen		†					†	
2- NOS Nordgau	†						†	
3- Peragis Garant			†					†
4- Heine’s Peko		†					†	
5- Hohenheimer Franken II	†					†		
6- Probat		†						†
7- Breustedt’s Lera		†					†	
8- Arin	†						†	
9- Kolibri	†				†			
10- Ralle	†					†		
11- Nandu				†				†
12- Fasan	†					†		
Number of genotypes per stages	6	4	1	1	1	3	5	3

† indicates the stages of visible floral degradation.

**Table 5.** Stages of visible floral degradation in 12 spring wheat cultivars grown in the greenhouse (control and tiller removal).

Cultivars	Control + Greenhouse				Detillering + Greenhouse			
	GA-YA	YA-TP	TP-HD	HD-AN	GA-YA	YA-TP	TP-HD	HD-AN
1- Adlung’s Alemannen		†					†	
2- NOS Nordgau		†					†	
3- Peragis Garant			†					†
4- Heine’s Peko		†					†	
5- Hohenheimer Franken II		†					†	
6- Probat			†					†
7- Breustedt’s Lera		†					†	
8- Arin			†				†	
9- Kolibri		†				†		
10- Ralle		†					†	
11- Nandu				†				†
12- Fasan			†					†
Number of genotypes per stages	0	7	4	1	0	1	7	4

† indicates the stages of visible floral degradation.

Generally, visible floral degradation in apical, central, or basal spikelets occurred simultaneously between the same two stages of the same genotypes. In the field, six free-tillering (i.e. control) genotypes underwent visible floral degradation between GA and YA, four between YA and TP, one between TP and HD, and one between HD and AN (Table 4); for detillered plants, one genotype underwent floral degradation in GA to YA, three in YA to TP, five in TP to HD, and three in HD to AN (Table 4). In the greenhouse, no control plant genotype underwent floral degradation in GA to YA, while seven showed degradation during YA to TP, four during TP to HD, and one during HD to AN (Table 5); for detillered plants, no genotypes underwent floral degradation from GA to YA, one showed degradation from YA to TP, seven from TP to HD, and four from HD to AN (Table 5).

After tiller removal, 11 genotypes grown in the field and 10 grown in the greenhouse showed delayed floral degradation (Tables 4 and 5). ‘Nandu’ did not show delayed floral degradation after tiller removal in either greenhouse or field conditions. In the greenhouse, besides ‘Nandu’, there was only one genotype (‘Arin’) that did not display delayed floral degradation after tiller removal (Table 5). ‘Nandu’ plants did not show delayed floral degradation after tiller removal in either field or greenhouse conditions because degradation occurred during HD to AN, which is the last possible stage for producing fertile florets (Tables 4 and 5).

Under greenhouse growth conditions, eight control genotypes demonstrated delayed floral degradation compared with field growth conditions. For the detillered treatment, only three genotypes (‘Peragis Garant’, ‘Probat’, and ‘Nandu’) exhibited delayed floral degradation because they were already in the last stage before fertile florets (Table 4).

### Influence of detillering on thermal time required for seven floral developmental stages

Although floral degradation was delayed by tiller removal and greenhouse conditions, tiller removal did not significantly affect the thermal time required for each floral developmental stage relative to controls (Supplementary Tables S9 and S10), with four exceptions. Exceptions included average thermal time required for GA (1069°Cd), YA (1121°Cd), and AN (1316°Cd) stages, which were significantly increased by tiller removal in the field (GA, 1104°Cd, *P* < 0.001; YA, 1149°Cd, *P* < 0.05; AN, 1349°Cd, *p* < 0.01) (Supplementary Table S9). Moreover, the average thermal time required for AN was significantly higher with tiller removal in the greenhouse (control, 1285°Cd; detillered, 1337°Cd; *P* < 0.05) (Supplementary Table S10). When considering specific genotypes, there were seven, six, and five cultivars at GA, YA, and AN stages, respectively, with significantly higher thermal time requirements after detillering in the field (Supplementary Table S9), and eight cultivars at AN stage after detillering in the greenhouse (Supplementary Table S10).

### Influence of detillering on maximum floret primordia number, fertile floret number, and final grain number per spikelet

**Fig. 5.**
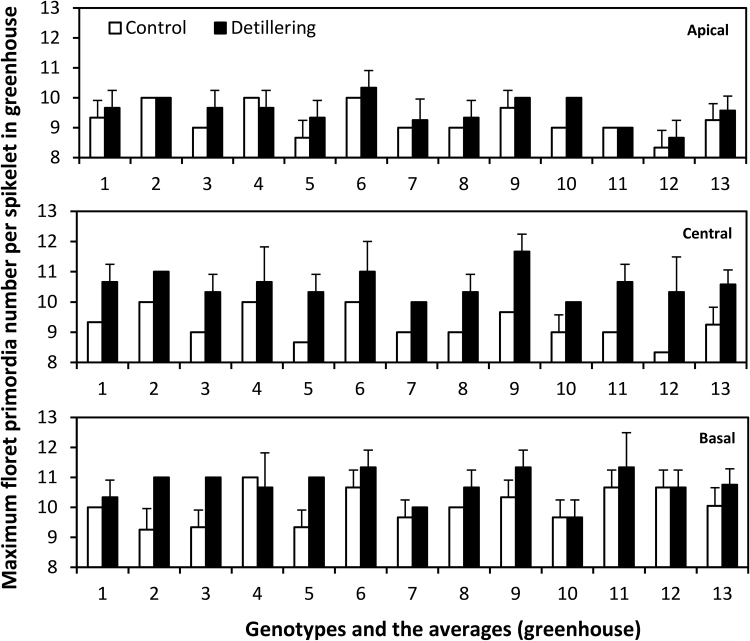
Maximum number of floret primordia per spikelet in apical, central, and basal positions in 12 genotypes (1–12) and averages of genotypes (13) under control and detillering treatments in the greenhouse (mean ± SD, n = 3).

Under greenhouse conditions, average maximum floret primordia number per spikelet was significantly lower for all 12 genotypes in control plants than in detillered plants at apical (control, 9.23; detillered, 9.60; *P* < 0.05), central (control, 10.20; detillered, 10.60; *P* < 0.05), and basal (control, 10.09; detillered, 10.77; *P* < 0.001) spikelet positions (Fig. 5). Under field conditions, average maximum floret primordia number per spikelet for all 12 genotypes was significantly lower in control plants than in detillered plants at apical (control, 9.72; detillered, 10.17; *P* < 0.01), central (control, 10.64; detillered, 10.94; *P* < 0.05), and basal (control, 10.67; detillered, 10.94; *P* < 0.1) spikelet positions (Supplementary Fig. S2). Generally, tiller removal increased the maximum floret primordia number at apical, central, and basal spikelets under both field and greenhouse conditions (Fig. 5 and Supplementary Fig. S2). The most obvious increase related to detillering, however, occurred at the central spikelet in the greenhouse (Fig. 5). Although maximum floret primordia number increased in most genotypes, it was relatively stable across positions, genotypes, treatments, and environments, with around 10 floret primordia, ranging from 9 to 12. This stability was in contrast to fertile floret number and final grain number. Central and basal spikelet positions generally had more floret primordia (Fig. 5).

**Fig. 6.**
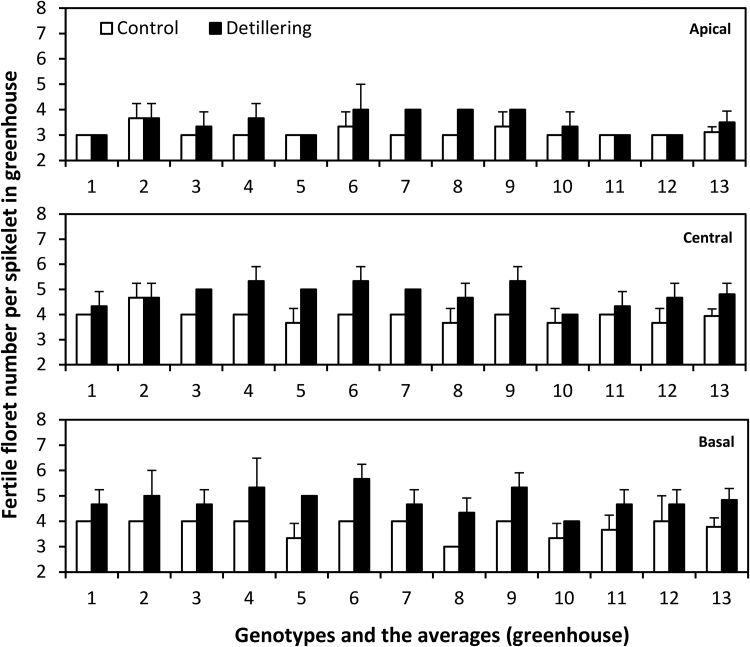
Fertile floret number per spikelet in apical, central, and basal positions in 12 (1–12) and averages of genotypes (13) under control and detillering treatments in greenhouse (mean ± SD, n = 3).

Under greenhouse conditions, average fertile floret number per spikelet for all 12 genotypes was significantly lower in control plants than in detillered plants at apical (control, 3.11; detillered, 3.51; *P* < 0.001), central (control, 3.94; detillered, 4.80; *P* < 0.001), and basal (control, 3.78; detillered, 4.80; *P* < 0.001) spikelet positions (Fig. 6). Under field conditions, average fertile floret number per spikelet for all 12 genotypes was significantly lower in control plants than in detillered plants at apical (control, 3.56; detillered, 4.00; *P* < 0.05), central (control, 4.69; detillered, 5.59; *P* < 0.001), and basal (control, 4.64; detillered, 5.56; *P* < 0.001) spikelets (Supplementary Fig. S3). Tiller removal significantly increased fertile floret numbers at apical, basal, and central positions in all 12 genotypes under both field and greenhouse conditions (Fig. 6 and Supplementary Fig. S3). The increase of fertile floret number was much more significant and consistent than for the maximum floret primordia number. However, there was strong fluctuation of fertile floret number across spikelet positions, genotypes, treatments, and environments (Fig. 6 and Supplementary Fig. S3), ranging from three to six. This indicates that fertile floret number per spikelet underlies positional effects within the spike. The significant increase and positional effects suggest large opportunities for improving fertile floret number, in particular for apical spikelets because central and basal spikelets generally had higher fertile floret number per spikelet (Fig. 6 and Supplementary Fig. S3).

**Fig. 7.**
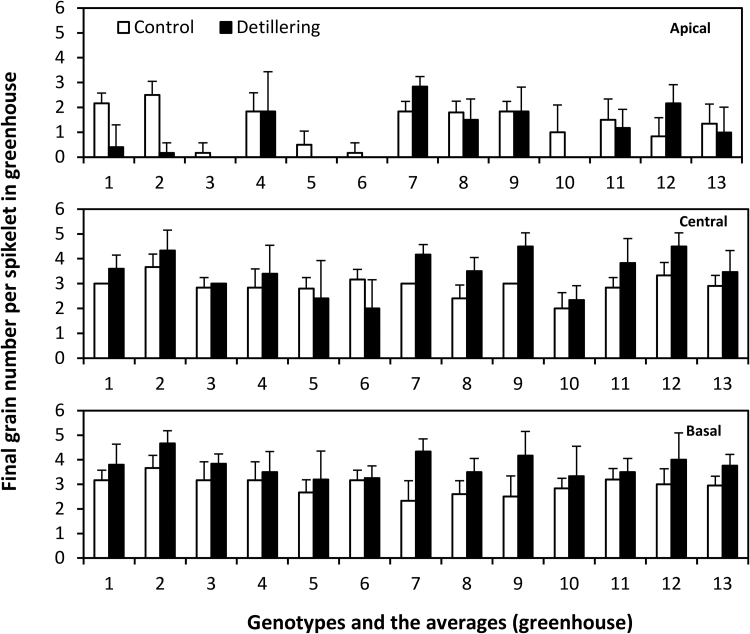
Final grain number per spikelet in apical, central and basal positions in 12 genotypes (1–12) and averages of genotypes (13) under control and detillering treatments in greenhouse (mean ± SD, n = 6).

For the average final grain number per spikelet for all 12 genotypes, a significant difference only occurred at basal spikelets in the greenhouse (control, 2.92; detillered, 3.78; *P* < 0.001) and at central spikelet positions in the field (control, 3.47; detillered, 3.82 detillered; *P* < 0.05) (Fig. 7 and Supplementary Fig. S4). In the greenhouse, there was no significant difference between control plants and detillered plants at the apical (control, 1.34; detillered, 1.04; *P* > 0.1) and central (control, 2.87; detillered, 3.24; *P* > 0.1) spikelets. In the field, there was no significant difference between control plants and detillered plants in the apical (control, 2.53; detillered, 2.25; *P* > 0.1) and basal (control, 3.49; detillered, 3.63; *P* > 0.1) spikelets (Fig. 7 and Supplementary Table S4). Generally, in most plants, the final grain number per spikelet was also increased by detillering under both greenhouse and field conditions (Fig. 7 and Supplementary Fig. S4). The most significant and consistent increase occurred in basal spikelets in the greenhouse where the final grain number per spikelet was markedly increased in all 12 genotypes (Fig. 7). The increase in final grain number per spikelet was not seen in apical spikelets in some genotypes of detillered plants. The lower apical spikelet fertility became more evident under the detillering treatment in the greenhouse (Fig. 7). The most important difference of final grain number per spikelet compared with maximum floret primordia and fertile floret number was that apical spikelets showed a large variation because, in some cultivars, apical spikelets did not set grain at all, which meant that the final grain number per spikelet was zero. It was concluded that the increase of final grain number per spikelet in basal and central spike positions was because more apical spikelets failed to set seeds owing to the competition between spikelets in different positions. Therefore, the large variation of final grain number and reduced spikelet fertility in apical spikelets was due to preferential resource allocation to the mid-bottom part of the spike.

### Influence of detillering on spikelet number, grain number per spike, and spikelet fertility

As shown in Table 6, detillering had no significant effects on total spikelet number per spike, but markedly decreased fertile spikelet number. This caused the considerable decrease of spikelet fertility (the ratio of fertile and total spikelet number). Under greenhouse conditions, the average spikelet fertility rate for all the 12 genotypes in control plants (78.50%) was markedly higher than in detillered plants (70.81%, *P* < 0.01). Similarly, under field conditions it was significantly higher in control plants (94.96%) than detillered plants (88.19%, *P* < 0.001). Grain number per spike was considerably increased by detillering in the greenhouse (control, 45.33; detillering, 53.34, *P* < 0.05), but not significantly influenced in the field. Generally, no association was found between spikelet fertility and year of release based on the 12 genotypes in this study (Supplementary Table S11). All these results suggest that the overall impact of detillering is complicated, because detillering increased grain number per spikelet in central and basal positions, but decreased the fertile spikelet number and spikelet fertility. In order to better understand the influence on grain number per spike and yield, the relationship between different traits should be studied.

**Table 6.** Total spikelet number, fertile spikelet number, spikelet fertility (%), grain number per spike, and grain number per spikelet (apical, central, basal positions) in 12 genotypes under control and tiller removal treatments under greenhouse and field conditions at harvest

Traits	Greenhouse		Field	
	Control	Detillering	Control	Detillering
Total spikelet number/spike	23.48±2.79^a^	23.29±2.69^a^	21.86±2.28^a^	21.53±2.25^a^
Fertile spikelet number/spike	18.24±3.02^a^	16.54±4.57^b^	20.77±2.63^a^	19.10±3.33^b^
Spikelet fertility (%)	78.50±15.16^a^	70.81±18.52^b^	94.96±6.05^a^	88.19±11.85^b^
Grain number/spike	45.33±9.16^b^	53.34±15.84^a^	59.18±17.21^a^	57.35±15.84^a^

Data are presented as the mean ±SD, n = 12; different letters per trait indicate significant differences between control and treated plants, P = 0.05.

## Discussion

Many previous studies have confirmed that tillers compete with the main shoot for resources (Mohamed and Marshall, 1979; Elalaoui *et al.*, 1988; Gu and Marshall, 1988). When tillers are removed, more assimilates become available to further enhance the growth potential of the main shoot. Here, leaf area (main shoot), spike dry weight (main shoot), and main stem dry weight at GA, AN, and PM were measured to determine the direct and indirect effects of tiller removal on the maximum number of floret primordia, fertile floret number, and final grain number (grain setting).

### Effects of tiller removal on the maximum floret primordia number per spikelet through growth of leaves, spikes, and stems on the main shoot

The maximum floret primordia number per spikelet stage was consistently found at the GA stage (Supplementary Tables S5, S6, S7, and S8). Hence, leaf area, spike dry weight, and stem dry weight of the main shoot were measured at this stage. As shown in Supplementary Fig. S5, main stem dry weight was significantly increased by tiller removal in both field and greenhouse conditions. Leaf area was also considerably increased by tiller removal under greenhouse conditions, but there was no significant difference between control and detillered plants in the field. In addition, there was a marked increase in spike dry weight after tiller removal in field-grown plants. Pre-anthesis stem dry mass accumulation has been documented to influence floral development and grain filling under stressed conditions (Bidinger *et al.*, 1977; Kiniry, 1993; Blum *et al.*, 1994; Blum, 1998). Thus, floret primordia may also benefit from an increase in main stem dry weight. Hence, these increases in the different structural parts of the main shoot can lead to increases in the maximum floret primordia number.

### Effects of tiller removal on fertile floret number per spikelet through growth of leaves, spikes, and stems on the main shoot

**Fig. 8.**
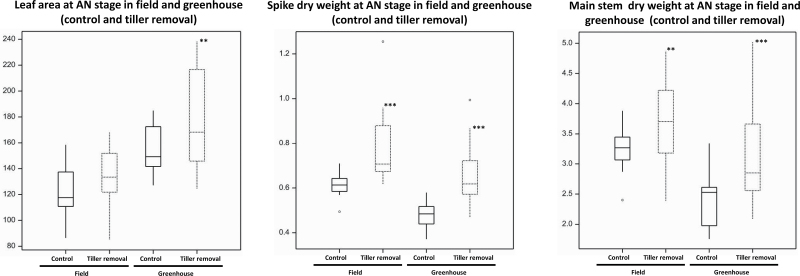
Boxplots of leaf area (cm^2^) (main shoot), spike dry weight (g) (main shoot), and main stem dry weight (g) at AN stage and significant levels of difference between control and tiller removal. **P < 0.01; ***P < 0.001. Each boxplot displays the average of the 12 genotypes for corresponding traits at AN stage; the significances suggest the comprehensive influence of detillering on different traits of the 12 genotypes.

Because the fertile floret number per spikelet was determined at AN, leaf area, spike dry weight, and stem dry weight of main shoot were also measured for this stage. Spike dry weight and stem dry weight of the main shoot were both markedly increased by tiller removal at AN in both field and greenhouse conditions. Leaf area was also considerably increased by tiller removal in greenhouse, but the increase was not very significant in field growth conditions (Fig. 8). Here, the situation for the number of fertile florets was similar to that of maximum floret primordia number at GA stage. After tiller removal, more resources may result in an increased number of fertile florets per spikelet. This is consistent with the work by Dreccer *et al.* (2014).

### Effects of tiller removal on final grain number per spikelet through growth of spikes and stems on the main shoot

Unlike for maximum floret primordia number and fertile floret number, some work has been done to understand the effects of detillering on final grain number per spike (grain setting). It was previously reported that detillering in wheat plants leads to more grains per spikelet. The increase in grain yield per spike was due to an increase in grain number per spikelet, particularly in the mid and lower spikelets of a spike (Mohamed and Marshall, 1979; Kemp and Whingwiri, 1980). Here, a substantial increase in main stem dry weight and spike chaff after tiller removal was found under both field and greenhouse conditions (Fig. 8), suggesting that the significant increase in spike chaff and main stem dry weight after detillering was responsible for the improvement of final grain number in the same environment.

### Effects of tiller removal on visible floral degradation by alleviating competition between tillers and the main shoot, spike, and stem

**Fig. 9.**
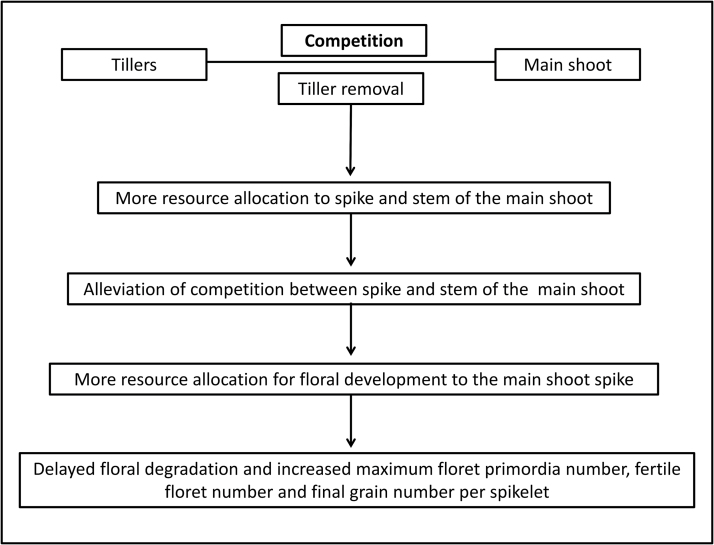
Effects of tiller removal on floral degradation, the maximum floret primordia number, fertile floret number, and final grain number by modifying competition between tillers and the main shoot, spike, and stem on main shoot.

Siddique *et al.* (1989) found an increase in grain number could be attributed to less competition from stem development. It was also reported that floret abortion occurs when the spike grows at its maximum rate (Fischer and Stockman, 1980; Kirby, 1988; González *et al.*, 2011). Because tillers compete with the main shoot for resources, and competition between the stem and spike is critical for determining grain number, detillering makes more resources available to spike and stem growth on the main shoot, which in turn can alleviate competition and thereby divert more resources towards floral development (Fig. 9). Furthermore, allocation of more resources toward floral development can delay visible floral degradation, resulting in one or two more floret primordia or final grains, respectively. Regrettably, more available resources cannot retain all developed floret primordia, and more than half of them still abort. Nevertheless, better understanding of the underlying developmental and resource-allocation–dependent limiting factors may shed more light on how to increase wheat’s yield potential in the future.

The Green Revolution refers to the vast increases in grain yields after the 1960s, resulting from the introduction of dwarfing genes, known as *Reduced height* (*Rht*) genes (Peng *et al.*, 1999; Hedden, 2003; Saville *et al.*, 2012). Dwarfing genes markedly enhanced the partitioning of dry matter to spikes’ growth in wheat (Abbate *et al.*, 1998; Fischer, 2007; Foulkes *et al.*, 2011). The thought is that shorter stems compete less with spikes for limited assimilate (Fischer and Stockman, 1986; Bancal, 2008).

Breeding strategies have been discussed and a lot of factors which may influence wheat yield have been considered (Reynolds *et al.*, 2009; Fischer, 2011; Foulkes *et al.*, 2011). The general idea is to improve assimilate partitioning to growing spikes. Because grain yield is more dependent upon grain number than on grain weight, increasing available assimilates to spike growth is an important target for wheat breeding (Reynolds *et al.*, 2009). Owing to the wheat breeding efforts over the past decades, the spike to stem ratio has been greatly increased (Siddique *et al.*, 1989; Slafer and Andrade, 1993; Siddique and Whan, 1994). Hence, the floret and spikelet fertility-related traits have undergone great improvement, and significantly contributed to wheat’s yield increase simply because more assimilates have been allocated to spikes (Fischer, 2011; Foulkes *et al.*, 2011). Lengthening stem elongation has been widely hypothesized to be an alternative approach to increase the number of fertile florets. Previous studies showed that when the duration of stem elongation increased under, for example, short photoperiod, the number of fertile florets was also increased owing to the increased partitioning to the spike at anthesis (Slafer, 1996; Miralles *et al.*, 2000; Alqudah and Schnurbusch, 2014). The data here suggest that delaying visible floral degradation may equally contribute to higher fertile floret numbers at anthesis. To what extent floral degradation is genetically or environmentally induced and independent of pre-anthesis phase durations remains to be shown.

Generally, around 6–12 floret primordia per spikelet initiated across the different spikelet positions within the spike; however, not more than usually five to six fertile florets can be found at anthesis (Langer and Hanif, 1973; Kirby, 1974, 1988; Ferrante et al., 2010, 2013). This is consistent with the presented findings (Supplementary Tables S5, S6, S7, and S8). The number of fertile florets at anthesis possibly depends upon two floral factors shaping the fate of apical floret primordia and more basal florets within a spikelet. One could be autophagy, regulated by day length or sugar supply, that might be involved in the abortion of apical floret primordia (Ghiglione *et al.*, 2008). Consistently, Dreccer *et al.* (2014) found that wheat lines with high spike biomass and sugar content at booting achieved more fertile florets. Whether autophagic processes are cause or consequence of apical floral abortion is not known. Development-dependent asynchronous floral progression between more basal florets (i.e. earliest florets) and younger apical floret primordia within spikelet may lead to less time for further differentiation processes, such as vascularization, in apical primordia. Thus, it is hypothesized that the vascular system within spikelets may limit the fertile floret number to a maximum of six. Consistent with the observations from this study, Hanif and Langer (1972) also found that only the first six florets (F1–F6) at the bottom of a spikelet were sufficiently connected by the vascular system; there was no vascular connectivity after F6. They showed that florets F1–F3 in the basal positions are supplied via the main vascular system from the rachilla, while the other three florets (F4–F6) share the branched-off veins from the main vasculature (Hanif and Langer, 1972). It seems that the final grain number per spikelet is predominantly associated with the connectivity of the main vascular system (F1–F3), while the fertile floret number is dependent upon the entirely developed vascular system (F4–F6). This observation should be further confirmed by studying the vascular system within spikelets.

## Supplementary data

Supplementary data are available at *JXB* online

Supplementary Table S1. Monthly average global solar radiation and temperature during the 2014 field growing season.

Supplementary Table S2. The general corresponding Waddington scales of F1 to the seven floral developmental stages based on the genotypes in this experiment.

Supplementary Table S3. Living floret primordia number in apical, central, and basal spikelets at seven floral developmental stages in the four selected free-tillering genotypes (control) grown in the greenhouse.

Supplementary Table S4. Living floret primordia number in apical, central, and basal spikelets at seven floral developmental stages in the four selected detillered genotypes (tiller removal) grown in the greenhouse.

Supplementary Table S5. Living floret primordia number in apical, central, and basal spikelet positions at seven floral developmental stages in 12 free-tillering genotypes (control) grown in the field.

Supplementary Table S6. Living floret primordia number in apical, central, and basal spikelet positions at seven floral developmental stages in 12 detillered genotypes (tiller removal) grown in the field.

Supplementary Table S7. Living floret primordia number in apical, central, and basal spikelet positions at seven floral developmental stages in 12 free-tillering genotypes (control) grown in the greenhouse.

Supplementary Table S8. Living floret primordia number in apical, central, and basal spikelet positions at seven floral developmental stages in 12 detillered genotypes (tiller removal) grown in the greenhouse.

Supplementary Table S9. Thermal time required for seven floral developmental stages in 12 spring wheat cultivars grown in the field (control and tiller removal).

Supplementary Table S10. Thermal time required for seven floral developmental stages in 12 spring wheat cultivars grown in the greenhouse (control and tiller removal).

Supplementary Table S11. Spikelet fertility (%) in 12 genotypes under control and tiller removal treatments in the greenhouse and field at harvest.

Supplementary Fig. S1. Control and tiller removal experiments in (A) field and (B) greenhouse.

Supplementary Fig. S2. Maximum floret primordia number per spike in apical, central, and basal spikelets in 12 genotypes (1–12) and averages of genotypes (13) under control and detillering treatments in field (mean ± SD, n = 3).

Supplementary Fig. S3. Fertile floret number per spike in apical, central, and basal spikelets in 12 genotypes (1–12) and averages of genotypes (13) under control and detillering treatments in field (mean ± SD, n = 3).

Supplementary Fig. S4. Final grain number per spike in apical, central, and basal spikelets in 12 genotypes (1–12) and averages of genotypes (13) under control and detillering treatments in field (mean ± SD, n = 6).

Supplementary Fig. S5. Boxplots of control and tiller removal for leaf area (cm^2^), leaf dry weight (g), spike dry weight (g), and main stem dry weight (g) at the GA stage and significant levels of difference between control and tiller removal.

Supplementary Fig. S6. Boxplots of main stem dry weight (g) and spike chaff (g) at PM stage and significant levels of difference between control and tiller removal.

Supplementary Data
