# Supplementary material for: Variation of floret fertility in hexaploid wheat revealed by tiller removal
Source: J Exp Bot. 2015 Jul 8;66(19):5945–58. doi: 10.1093/jxb/erv303 (PMC4566983; doi:10.1093/jxb/erv303)
Supplement: Supplementary Data [file supp_66_19_5945__index.html]

Variation of floret fertility in hexaploid wheat revealed by tiller removal — Supplementary Data 

# Variation of floret fertility in hexaploid wheat revealed by tiller removal

## Supplementary Data

Data files

- Supplementary Data - Supplementary Data
