# Supplementary material for: Variation of floret fertility in hexaploid wheat revealed by tiller removal
Source: J Exp Bot. 2015 Jul 8;66(19):5945–58. doi: 10.1093/jxb/erv303 (PMC4566983; doi:10.1093/jxb/erv303)
Supplement: Supplementary Data [file supp_erv303_2015_07_06_Supplementary_materials_JXB.docx]

**Supplementary materials**

**Table S1.** Monthly average global solar radiation and temperature during the 2014 field growing season.

| Climatic data\Month | April | May | June | July | August |
| --- | --- | --- | --- | --- | --- |
| Global solar radiation (W/m^2^) | 169.2 | 191.2 | 222.8 | 208.5 | 173.7 |
| Temperature (^o^C) | 11.4 | 12.9 | 16.3 | 20.3 | 16.6 |

**Table S2.** The corresponding Waddington stages of floret1 (i.e. F1) at the seven floral developmental stages based on the phenotypic variation observed in this experiment.

| Kirby scale | TS | WA | GA | YA | TP | HD | AN |
| --- | --- | --- | --- | --- | --- | --- | --- |
| Waddington scale | 4-5 | 7-7.5 | 7.5-8.5 | 8-8.5 | 8.5-9 | 9-9.5 | 10 |

**Table S3.** Living floret (primordia) number per spikelet in apical, central and basal spikelets at seven floral developmental stages and grain number per spikelet at physiological maturity (PM) in the four selected, free-tillering genotypes (control) grown in the greenhouse.

| **Cultivars** | **Position** | **TS** | **WA** | **GA** | **YA** | **TP** | **HD** | **AN** | **PM** |
| --- | --- | --- | --- | --- | --- | --- | --- | --- | --- |
| NOS Nordgau | Apical | 3.33±1.53 | 7.67±1.15 | **9.33±0.58** | 9.00±0.00 | 3.00±0.00 | 3.67±0.58 | 3.00±0.00 | 2.17±0.41 |
|  | Central | 5.00±1.00 | 9.33±1.15 | **10.00±0.00** | 9.67±0.58 | 4.00±0.00 | 4.33±0.58 | 4.00±0.00 | 3.00±0.00 |
|  | Basal | 5.67±1.53 | 9.33±1.15 | **10.00±0.00** | 10.00±0.00 | 4.00±0.00 | 4.33±0.58 | 4.00±0.00 | 3.17±0.41 |
| Adlung’s Alemannen | Apical | 4.33±1.15 | 9.00±1.00 | **10.00±0.0** | 9.67±0.58 | 4.00±0.00 | 4.00±0.00 | 3.67±0.58 | 2.50±0.55 |
|  | Central | 5.00±1.00 | 8.67±0.58 | **10.00±0.00** | 11.00±0.00 | 4.00±0.00 | 4.67±0.58 | 4.67±0.58 | 3.67±0.52 |
|  | Basal | 5.00±1.00 | 8.33±0.58 | **9.50±0.71** | 10.33±1.00 | 4.00±0.00 | 4.33±0.58 | 4.00±0.00 | 3.67±0.52 |
| Peragis Garant | Apical | 4.00±1.00 | 7.50±0.71 | **10.00±0.00** | 10.00±0.00 | 9.00±0.00 | 4.00±0.00 | 3.00±0.58 | 1.83±0.75 |
|  | Central | 6.33±0.58 | 8.00±0.00 | **11.00±0.00** | 10.00±1.00 | 9.67±0.58 | 5.00±0.00 | 4.00±0.58 | 2.83±0.75 |
|  | Basal | 7.33±0.58 | 10.00±1.41 | **11.00±0.00** | 10.67±0.58 | 10.00±0.00 | 5.33±0.58 | 4.000.00 | 3.17±0.75 |
| Nandu | Apical | 5.00±0.00 | 9.50±0.71 | **9.67±0.58** | 10.00±0.00 | 9.67±0.58 | 9.33±0.58 | 3.33±0.58 | 1.83±0.41 |
|  | Central | 5.67±0.58 | 10.00±0.00 | **11.00±0.00** | 11.00±0.00 | 10.33±0.58 | 10.00±0.00 | 4.00±0.00 | 3.00±0.00 |
|  | Basal | 6.33±1.53 | 10.50±0.71 | **10.33±0.58** | 10.33±0.58 | 11.33±0.58 | 11.33±0.58 | 4.00±0.00 | 2.50±0.84 |

Data are presented as the mean [±](http://www.baidu.com/link?url=duy-wQqaYmgEkMTManTS8fcdGS-xbMeKDPsL9DSBR6AwBaLBFd1-kWBE4IDWU3glXrNYbHrT81vdNQf8u8feiq)SD, n=6 for PM stage, and n=3 for the remaining stages. The bold text suggests maximum floret primordia number stage is GA stage, and the grey boxes indicate the time windows of floral degradation occrued.

**Table S4.** Living floret (primordia) number per spikelet in apical, central and basal spikelets at seven floral developmental stages and grain number per spikelet at physiological maturity (PM) in the four selected, detillered genotypes (tiller removal) grown in the greenhouse.

| **Cultivars** | **Position** | **TS** | **WA** | **GA** | **YA** | **TP** | **HD** | **AN** | **PM** |
| --- | --- | --- | --- | --- | --- | --- | --- | --- | --- |
| NOS Nordgau | Apical | 4.00±1.00 | 9.00±0.00 | **9.67±0.58** | 9.33±0.58 | 9.33±0.58 | 3.67±0.58 | 3.00±0.00 | 0.40±0.89 |
|  | Central | 5.00±1.00 | 9.50±0.71 | **10.67±0.58** | 11.00±0.00 | 11.00±0.00 | 4.67±0.58 | 4.33±0.58 | 3.60±0.55 |
|  | Basal | 5.00±1.00 | 10.00±1.41 | **10.33±0.58** | 10.33±0.58 | 11.33±0.58 | 5.00±0.00 | 4.67±0.58 | 3.80±0.84 |
| Adlung’s Alemannen | Apical | 3.33±0.58 | 9.00±0.00 | **10.00±0.00** | 10.00±0.00 | 10.00±0.00 | 4.00±0.00 | 3.67±0.58 | 0.17±0.41 |
|  | Central | 4.33±0.58 | 9.33±0.58 | **11.00±0.00** | 11.00±0.00 | 10.00±0.00 | 5.00±0.00 | 4.67±0.58 | 4.33±0.82 |
|  | Basal | 4.67±0.58 | 9.00±0.00 | **11.00±0.00** | 11.00±0.00 | 10.67±0.58 | 5.00±1.00 | 5.00±1.00 | 4.67±0.52 |
| Peragis Garant | Apical | 5.00±2.00 | 8.33±1.15 | **9.67±0.58** | 10.00±0.00 | 10.00±0.00 | 10.00±0.00 | 3.67±0.58 | 1.83±1.60 |
|  | Central | 6.67±1.53 | 9.00±0.00 | **10.67±1.15** | 11.67±0.58 | 11.00±0.00 | 11.33±0.58 | 5.33±0.58 | 2.83±1.72 |
|  | Basal | 7.33±1.15 | 9.67±1.15 | **10.67±1.15** | 10.67±0.58 | 11.00±0.00 | 11.00±0.00 | 5.33±1.15 | 3.50±0.84 |
| Nandu | Apical | 4.33±1.15 | 9.67±0.58 | **10.00±0.00** | 9.67±1.15 | 10.33±0.58 | 10.67±0.58 | 4.00±0.00 | 1.83±0.98 |
|  | Central | 5.33±0.58 | 10.33±1.15 | **11.67±0.58** | 11.33±0.58 | 11.67±0.58 | 11.33±1.53 | 5.33±0.58 | 4.50±0.55 |
|  | Basal | 4.67±0.58 | 10.67±0.58 | **11.33±0.58** | 11.33±0.58 | 11.67±0.58 | 12.00±0.00 | 5.33±0.58 | 4.17±0.98 |

Data are presented as the mean [±](http://www.baidu.com/link?url=duy-wQqaYmgEkMTManTS8fcdGS-xbMeKDPsL9DSBR6AwBaLBFd1-kWBE4IDWU3glXrNYbHrT81vdNQf8u8feiq) SD, n=6 for PM stage, and n=3 for the remaining stages. The bold text suggests maximum floret primordia number stage is GA stage, and the grey boxes indicate the time windows of floral degradation occrued.

**Table S5.** Living floret (primordia) number per spikelet in apical, central and basal spikelets at seven floral developmental stages and grain number per spikelet at physiological maturity (PM) in twelve free-tillering genotypes (control) grown in the field.

| **Cultivars (control, field)** | **Position** | **TS** | **WA** | **GA** | **YA** | **TP** | **HD** | **AN** | **PM** |
| --- | --- | --- | --- | --- | --- | --- | --- | --- | --- |
| 01- Adlung’s Alemannen | Apical | 2.33±0.58 | 9.00±1.00 | **9.67±0.58** | 9.67±1.15 | 4.33±0.58 | 4.33±0.58 | 3.00±0.00 | 2.50±0.55 |
|  | Central | 3.33±0.58 | 10.00±0.00 | **10.67±0.58** | 10.00±0.00 | 5.33±0.58 | 5.33±0.58 | 4.33±0.58 | 3.67±0.82 |
|  | Basal | 3.33±0.58 | 9.33±0.58 | **10.33±0.58** | 11.00±0.00 | 5.33±0.58 | 5.00±1.00 | 4.33±0.58 | 3.50±0.55 |
| 2- NOS Nordgau | Apical | 2.00±0.00 | 8.67±0.58 | **10.00±0.00** | 4.00±0.00 | 4.00±0.00 | 4.00±0.00 | 3.00±0.00 | 2.83±0.75 |
|  | Central | 3.67±0.58 | 9.00±0.00 | **10.33±0.58** | 5.67±0.58 | 5.00±0.00 | 5.00±0.00 | 4.33±0.58 | 3.67±0.82 |
|  | Basal | 3.00±0.00 | 9.67±0.58 | **10.33±0.58** | 5.67±0.58 | 5.00±0.00 | 5.00±0.00 | 4.33±0.58 | 3.67±0.52 |
| 3- Peragis Garant | Apical | 4.00±0.00 | 8.67±1.15 | **10.00±0.00** | 9.33±1.15 | 9.67±1.15 | 4.00±0.00 | 3.67±0.58 | 2.67±0.52 |
|  | Central | 5.33±0.58 | 10.33±0.58 | **11.00±1.00** | 11.00±1.00 | 10.67±1.15 | 5.67±0.58 | 5.00±1.00 | 3.50±0.84 |
|  | Basal | 5.33±0.58 | 10.33±0.58 | **11.33±0.58** | 10.67±0.58 | 11.67±0.58 | 6.00±0.00 | 5.00±1.00 | 3.00±1.26 |
| 4- Heine’s Peko | Apical | 2.67±0.58 | 8.00±1.00 | **9.33±0.58** | 9.00±0.00 | 4.00±0.00 | 3.00±0.00 | 3.00±0.00 | 1.83±0.41 |
|  | Central | 4.00±1.00 | 9.67±0.58 | **10.33±0.58** | 10.33±0.58 | 5.00±0.00 | 3.67±0.58 | 4.00±0.00 | 2.67±0.52 |
|  | Basal | 3.67±0.58 | 9.67±0.58 | **10.67±0.58** | 10.33±0.58 | 5.00±0.00 | 3.67±0.58 | 4.00±0.00 | 2.67±0.52 |
| 5- Hohenheimer Franken II | Apical | 3.33±0.58 | 9.00±1.00 | **10.00±0.00** | 4.33±0.58 | 4.00±0.00 | 3.33±0.58 | 3.33±0.58 | 2.33±0.52 |
|  | Central | 4.00±1.00 | 9.00±0.00 | **10.33±0.58** | 5.00±0.00 | 4.67±0.58 | 4.00±0.00 | 4.00±0.00 | 2.50±0.55 |
|  | Basal | 3.33±0.58 | 9.00±0.00 | **10.00±0.00** | 4.67±0.58 | 4.67±0.58 | 4.00±0.00 | 4.00±0.00 | 3.00±0.63 |
| 6- Probat | Apical | 3.33±0.58 | 9.33±0.58 | **10.00±0.00** | 9.67±1.15 | 6.00±3.46 | 5.00±0.00 | 4.33±0.58 | 2.50±0.55 |
|  | Central | 5.33±0.58 | 10.67±0.58 | **11.00±0.00** | 11.67±0.58 | 7.67±2.89 | 6.00±0.00 | 6.00±0.00 | 3.83±0.75 |
|  | Basal | 4.33±0.58 | 11.00±0.00 | **11.00±0.00** | 11.00±1.00 | 7.33±3.21 | 6.00±0.00 | 5.33±0.58 | 3.33±1.03 |
| 7- Breustedt’s Lera | Apical | 2.67±0.58 | 8.33±0.58 | **9.67±0.58** | 9.33±0.58 | 4.33±0.58 | 4.00±0.00 | 4.00±0.00 | 2.33±1.37 |
|  | Central | 5.00±1.00 | 9.67±0.58 | **11.00±0.00** | 10.67±0.58 | 4.67±0.58 | 5.33±1.15 | 5.33±0.58 | 3.83±1.47 |
|  | Basal | 4.67±0.58 | 9.00±0.00 | **10.67±0.58** | 11.00±0.00 | 4.67±0.58 | 5.00±1.00 | 5.00±0.00 | 4.50±0.55 |
| 8- Arin | Apical | 3.67±0.58 | 9.00±0.00 | **9.33±0.58** | 5.00±0.00 | 5.00±0.00 | 4.67±0.58 | 4.00±0.00 | 3.50±1.05 |
|  | Central | 5.33±0.58 | 9.67±0.58 | **10.67±0.58** | 6.00±0.00 | 6.00±0.00 | 6.00±0.00 | 5.00±0.00 | 4.17±0.75 |
|  | Basal | 5.67±0.58 | 10.67±0.58 | **11.00±0.00** | 6.00±0.00 | 6.00±0.00 | 6.00±0.00 | 5.00±0.00 | 4.50±0.84 |
| 9- Kolibri | Apical | 4.00±1.00 | 8.50±0.71 | **9.00±1.00** | 4.00±0.00 | 3.33±0.58 | 4.00±0.00 | 3.67±0.58 | 2.33±0.52 |
|  | Central | 5.00±1.00 | 9.00±0.00 | **10.00±0.00** | 4.67±0.58 | 4.33±0.58 | 4.67±0.58 | 4.67±0.58 | 3.17±0.75 |
|  | Basal | 4.67±1.15 | 9.00±0.00 | **10.00±0.00** | 4.00±0.00 | 4.33±0.58 | 5.00±1.00 | 4.67±0.58 | 3.33±1.03 |
| 10- Ralle | Apical | 4.00±1.00 | 8.67±0.58 | **9.00±0.00** | 4.33±0.58 | 4.00±0.00 | 4.00±0.00 | 3.67±0.58 | 2.17±0.41 |
|  | Central | 5.67±0.58 | 9.67±0.58 | **10.00±0.00** | 5.00±0.00 | 5.00±0.00 | 5.00±0.00 | 4.33±1.15 | 3.17±0.41 |
|  | Basal | 5.00±1.00 | 9.33±0.58 | **10.00±0.00** | 5.33±0.58 | 5.00±0.00 | 4.67±0.58 | 4.00±1.00 | 2.83±0.75 |
| 11- Nandu | Apical | 3.33±0.58 | 9.00±0.00 | **10.67±0.58** | 10.33±0.58 | 10.00±0.00 | 10.00±0.00 | 3.33±0.58 | 2.50±0.55 |
|  | Central | 5.33±0.58 | 10.00±0.00 | **11.67±0.58** | 11.33±0.58 | 11.00±0.00 | 11.00±0.00 | 4.33±0.58 | 3.67±0.52 |
|  | Basal | 5.00±1.00 | 10.00±0.00 | **11.67±0.58** | 12.00±0.00 | 11.67±0.58 | 12.00±1.00 | 4.33±0.58 | 3.33±1.03 |
| 12- Fasan | Apical | 2.67±0.58 | 8.67±0.58 | **10.00±0.00** | 4.67±0.58 | 4.33±0.58 | 4.00±0.00 | 3.67±0.58 | 2.83±0.41 |
|  | Central | 4.33±1.15 | 10.33±0.58 | **10.67±0.58** | 6.00±0.00 | 5.33±0.58 | 5.00±0.00 | 5.00±0.00 | 3.83±1.17 |
|  | Basal | 4.33±1.15 | 10.33±0.58 | **11.00±0.00** | 6.00±0.00 | 6.00±0.00 | 5.33±0.58 | 5.67±0.58 | 4.17±1.47 |

Data are presented as the mean [±](http://www.baidu.com/link?url=duy-wQqaYmgEkMTManTS8fcdGS-xbMeKDPsL9DSBR6AwBaLBFd1-kWBE4IDWU3glXrNYbHrT81vdNQf8u8feiq) SD, n=6 for PM stage, and n=3 for the remaining stages. The bold text suggests maximum floret primordia number stage is GA stage, and the grey boxes indicate the time windows of floral degradation occrued.

**Table S6.** Living floret (primordia) number per spikelet in apical, central and basal spikelets at seven floral developmental stages and grain number per spikelet at physiological maturity (PM) in twelve detillered genotypes (tiller removal) grown in the field.

| **Cultivars (detillering, field)** | **Position** | **TS** | **WA** | **GA** | **YA** | **TP** | **HD** | **AN** | **PM** |
| --- | --- | --- | --- | --- | --- | --- | --- | --- | --- |
| 1- Adlung’s Alemannen | Apical | 3.50±0.71 | 8.67±0.58 | **10.67±0.58** | 10.67±0.58 | 10.33±0.58 | 5.67±0.58 | 5.00±1.00 | 2.33±1.75 |
|  | Central | 4.50±0.71 | 9.67±0.58 | **10.67±0.58** | 11.33±0.58 | 11.67±0.58 | 6.00±1.00 | 6.00±0.00 | 4.67±0.52 |
|  | Basal | 4.00±0.00 | 9.67±0.58 | **11.00±0.00** | 11.33±0.58 | 12.00±0.00 | 5.67±0.58 | 6.00±0.00 | 4.33±1.21 |
| 2- NOS Nordgau | Apical | 2.00±0.00 | 8.33±0.58 | **10.67±0.58** | 10.33±0.58 | 9.67±0.58 | 5.67±0.58 | 3.67±0.58 | 3.00±1.55 |
|  | Central | 3.33±0.58 | 9.00±1.00 | **10.67±0.58** | 11.00±0.00 | 11.00±1.00 | 6.33±0.58 | 6.00±0.00 | 4.50±0.84 |
|  | Basal | 3.33±0.58 | 9.67±0.58 | **10.67±0.58** | 10.67±0.58 | 11.00±0.00 | 6.00±0.00 | 6.00±0.00 | 4.50±0.84 |
| 3- Peragis Garant | Apical | 4.00±1.73 | 9.67±0.58 | **10.67±0.58** | 10.67±0.58 | 10.67±0.58 | 9.00±3.46 | 3.33±0.58 | 2.50±1.05 |
|  | Central | 5.33±0.58 | 10.33±0.58 | **11.67±0.58** | 12.00±0.00 | 11.33±0.58 | 10.00±3.46 | 5.00±0.00 | 3.33±1.03 |
|  | Basal | 6.67±0.58 | 9.67±0.58 | **11.67±0.58** | 12.00±0.00 | 12.33±0.58 | 10.00±3.46 | 5.00±0.00 | 3.33±1.03 |
| 4- Heine’s Peko | Apical | 2.00±0.00 | 8.33±0.58 | **9.33±0.58** | 9.67±0.58 | 9.67±0.58 | 4.00±0.00 | 3.67±0.58 | 2.00±0.63 |
|  | Central | 4.00±1.00 | 9.33±1.15 | **11.00±0.00** | 10.67±0.58 | 10.33±0.58 | 5.67±0.58 | 5.67±0.58 | 3.50±0.84 |
|  | Basal | 4.00±1.00 | 9.33±0.58 | **11.00±0.00** | 11.33±0.58 | 11.33±0.58 | 5.67±0.58 | 5.67±0.58 | 3.33±1.03 |
| 5- Hohenheimer Franken II | Apical | 2.33±0.58 | 9.33±1.53 | **10.00±0.00** | 10.00±0.00 | 5.00±0.00 | 4.00±0.00 | 4.33±0.58 | 0.50±0.55 |
|  | Central | 3.00±1.00 | 9.67±0.58 | **10.67±0.58** | 11.00±0.00 | 5.00±0.00 | 5.00±0.00 | 5.33±0.58 | 3.33±0.52 |
|  | Basal | 2.33±0.58 | 9.67±1.53 | **10.00±0.00** | 11.00±0.00 | 5.00±0.00 | 5.00±0.00 | 5.00±0.00 | 3.50±0.55 |
| 6- Probat | Apical | 3.00±0.00 | 8.67±0.58 | **10.33±0.58** | 11.00±0.00 | 11.00±0.00 | 11.00±0.00 | 5.00±2.83 | 2.25±0.96 |
|  | Central | 5.00±0.00 | 9.67±0.58 | **11.67±0.58** | 12.50±0.71 | 12.00±0.00 | 12.00±0.00 | 6.00±1.41 | 4.00±1.15 |
|  | Basal | 4.00±1.41 | 10.67±0.58 | **11.67±0.58** | 12.00±0.00 | 12.00±0.00 | 12.00±0.00 | 6.50±0.71 | 3.50±1.29 |
| 7- Breustedt’s Lera | Apical | 2.33±0.58 | 8.67±0.58 | **10.33±0.58** | 10.00±0.00 | 9.50±0.71 | 4.50±0.71 | 4.00±1.41 | 2.00±1.73 |
|  | Central | 4.00±1.00 | 10.33±1.15 | **11.33±0.58** | 11.50±0.71 | 11.50±0.71 | 5.50±0.71 | 6.00±0.00 | 4.33±0.58 |
|  | Basal | 4.00±1.00 | 10.67±0.58 | **11.33±0.58** | 12.00±1.41 | 12.00±0.00 | 5.50±0.71 | 6.00±0.00 | 3.33±1.53 |
| 8- Arin | Apical | 3.33±0.58 | 9.00±0.00 | **9.67±0.58** | 9.00±0.00 | 9.00±0.00 | 4.67±0.58 | 4.33±0.58 | 3.50±1.00 |
|  | Central | 4.67±0.58 | 10.00±0.00 | **10.33±0.58** | 11.00±0.00 | 10.50±0.71 | 5.67±0.58 | 5.00±0.00 | 3.75±1.26 |
|  | Basal | 6.00±1.00 | 9.67±1.15 | **10.00±1.00** | 10.33±1.15 | 11.00±0.00 | 6.67±0.58 | 5.67±0.58 | 3.75±0.96 |
| 9- Kolibri | Apical | 3.33±0.58 | 9.00±0.00 | **9.67±0.58** | 4.67±0.58 | 4.33±0.58 | 4.00±0.00 | 3.33±0.58 | 2.83±0.41 |
|  | Central | 4.67±0.58 | 9.67±0.58 | **10.33±0.58** | 5.67±0.58 | 5.67±0.58 | 5.67±0.58 | 5.67±0.58 | 4.17±1.17 |
|  | Basal | 4.00±1.00 | 9.67±0.58 | **10.33±0.58** | 5.67±0.58 | 5.67±0.58 | 5.67±0.58 | 5.33±0.58 | 3.50±1.52 |
| 10- Ralle | Apical | 3.33±0.58 | 9.00±0.00 | **9.67±0.58** | 9.33±0.58 | 5.00±0.00 | 4.00±0.00 | 3.67±0.58 | 1.50±1.64 |
|  | Central | 5.33±0.58 | 9.67±0.58 | **11.00±0.00** | 10.33±0.58 | 6.00±0.00 | 6.00±0.00 | 4.67±0.58 | 3.33±0.82 |
|  | Basal | 4.67±0.58 | 10.00±0.00 | **11.00±1.00** | 12.00±0.00 | 6.00±0.00 | 7.33±2.31 | 4.67±0.58 | 4.00±0.89 |
| 11- Nandu | Apical | 3.67±1.15 | 8.00±0.00 | **10.67±0.58** | 11.00±0.00 | 11.00±0.00 | 11.00±1.00 | 4.67±0.58 | 3.29±0.00 |
|  | Central | 5.67±0.58 | 9.00±0.00 | **11.00±0.00** | 12.33±0.58 | 12.00±0.00 | 12.33±0.58 | 6.67±0.58 | 4.00±0.75 |
|  | Basal | 6.00±1.00 | 10.00±0.00 | **11.33±0.58** | 11.67±0.58 | 12.33±0.58 | 13.00±0.00 | 6.67±0.58 | 3.43±0.98 |
| 12- Fasan | Apical | 3.00±0.00 | 9.00±0.00 | **10.33±0.58** | 10.00±0.00 | 4.00±0.00 | 4.33±0.58 | 3.33±0.58 | 1.83±1.33 |
|  | Central | 5.00±0.00 | 10.00±0.00 | **11.00±0.00** | 11.00±0.00 | 5.00±0.00 | 5.67±0.58 | 5.00±0.00 | 3.00±0.63 |
|  | Basal | 5.00±0.00 | 9.67±0.58 | **11.33±0.58** | 12.00±0.00 | 5.67±0.58 | 5.67±0.58 | 5.00±1.00 | 3.17±0.98 |

Data are presented as the mean [±](http://www.baidu.com/link?url=duy-wQqaYmgEkMTManTS8fcdGS-xbMeKDPsL9DSBR6AwBaLBFd1-kWBE4IDWU3glXrNYbHrT81vdNQf8u8feiq) SD, n=6 for PM stage, and n=3 for the remaining stages. The bold text suggests maximum floret primordia number stage is GA stage, and the grey boxes indicate the time windows of floral degradation occrued.

**Table S7.** Living floret (primordia) number per spikelet in apical, central and basal spikelets at seven floral developmental stages and grain number per spikelet at physiological maturity (PM) in twelve free-tillering genotypes (control) grown in the greenhouse.

| **Cultivars (control, greenhouse)** | **Position** | **TS** | **WA** | **GA** | **YA** | **TP** | **HD** | **AN** | **PM** |
| --- | --- | --- | --- | --- | --- | --- | --- | --- | --- |
| 1- Adlung’s Alemannen | Apical | 4.33±1.15 | 9.00±1.00 | **10.00±0.00** | 9.67±0.58 | 4.00±0.00 | 4.00±0.00 | 3.67±0.58 | 2.50±0.55 |
|  | Central | 5.00±1.00 | 8.67±0.58 | **10.00±0.00** | 11.00±0.00 | 4.00±0.00 | 4.67±0.58 | 4.67±0.58 | 3.67±0.52 |
|  | Basal | 5.00±1.00 | 8.33±0.58 | **9.50±0.71** | 10.33±1.15 | 4.00±0.00 | 4.33±0.58 | 4.00±0.00 | 3.67±0.52 |
| 2- NOS Nordgau | Apical | 3.33±1.53 | 7.67±1.15 | **9.33±0.00** | 9.00±0.00 | 3.00±0.00 | 3.67±0.58 | 3.00±0.00 | 2.17±0.41 |
|  | Central | 5.00±1.00 | 9.33±1.15 | **10.00±0.00** | 9.67±0.58 | 4.00±0.00 | 4.33±0.58 | 4.00±0.00 | 3.00±0.00 |
|  | Basal | 5.67±1.53 | 9.33±1.15 | **10.00±0.00** | 10.00±0.00 | 4.00±0.00 | 4.33±0.58 | 4.00±0.00 | 3.17±0.41 |
| 3- Peragis Garant | Apical | 4.00±1.00 | 7.50±0.71 | **10.00±0.00** | 10.00±0.00 | 9.00±0.00 | 4.00±0.00 | 3.00±0.00 | 1.83±0.75 |
|  | Central | 6.33±0.58 | 8.00±0.00 | **11.00±0.00** | 10.00±1.00 | 9.67±0.58 | 5.00±0.00 | 4.00±0.00 | 2.83±0.75 |
|  | Basal | 7.33±0.58 | 10.00±1.41 | **11.00±0.00** | 10.67±0.58 | 10.00±0.00 | 5.33±0.58 | 4.00±0.00 | 3.17±0.75 |
| 4- Heine’s Peko | Apical | 2.33±0.58 | 7.33±0.58 | **9.00±0.00** | 9.33±0.58 | 3.00±0.00 | 3.00±0.00 | 3.00±0.00 | 0.17±0.41 |
|  | Central | 4.67±0.58 | 8.67±0.58 | **10.00±0.00** | 10.00±0.00 | 4.00±0.00 | 4.00±0.00 | 4.00±0.00 | 2.83±0.41 |
|  | Basal | 4.33±0.58 | 8.33±0.58 | **9.33±0.58** | 9.67±0.58 | 3.67±0.58 | 4.00±0.00 | 4.00±0.00 | 3.17±0.75 |
| 5- Hohenheimer Franken II | Apical | 2.00±0.00 | 8.33±0.58 | **9.00±0.00** | 9.00±0.00 | 3.33±0.58 | 3.00±0.00 | 3.00±0.00 | 1.00±1.10 |
|  | Central | 2.67±0.58 | 8.33±0.58 | **10.33±0.58** | 9.00±0.00 | 4.00±0.00 | 4.00±0.00 | 3.67±0.58 | 2.00±0.63 |
|  | Basal | 2.33±0.58 | 8.67±0.58 | **9.67±0.58** | 9.33±0.58 | 4.00±0.00 | 4.00±0.00 | 3.33±0.58 | 2.83±0.41 |
| 6- Probat | Apical | 3.67±0.58 | 7.67±1.15 | **10.00±0.00** | 10.00±1.00 | 9.33±0.58 | 4.00±0.00 | 3.33±0.58 | 0.17±0.41 |
|  | Central | 6.00±0.00 | 10.33±0.58 | **11.00±0.00** | 11.00±1.00 | 10.00±1.00 | 4.67±0.58 | 4.00±0.00 | 3.17±0.41 |
|  | Basal | 5.67±0.58 | 9.67±0.58 | **10.67±0.58** | 11.00±1.00 | 11.33±0.58 | 4.67±0.58 | 4.00±0.00 | 3.17±0.41 |
| 7- Breustedt’s Lera | Apical | 3.00±1.00 | 8.00±0.00 | **8.67±0.58** | 8.67±0.58 | 3.33±0.58 | 3.67±0.58 | 3.00±0.00 | 0.50±0.55 |
|  | Central | 5.67±1.15 | 8.67±0.58 | **10.00±0.00** | 10.00±0.00 | 4.67±0.58 | 4.67±0.58 | 3.67±0.58 | 2.33±1.21 |
|  | Basal | 5.33±1.53 | 9.33±0.58 | **9.33±0.58** | 9.33±0.58 | 4.67±0.58 | 4.67±0.58 | 3.33±0.58 | 2.67±0.52 |
| 8- Arin | Apical | 4.00±0.00 | 7.67±0.58 | **8.33±0.58** | 8.67±0.58 | 9.00±0.00 | 3.67±0.58 | 3.00±0.00 | 0.83±0.75 |
|  | Central | 5.67±0.58 | 8.67±0.58 | **9.00±0.00** | 9.00±0.00 | 9.00±0.58 | 5.00±0.00 | 3.67±0.58 | 3.33±0.52 |
|  | Basal | 5.67±1.15 | 9.00±0.00 | **10.67±0.58** | 10.33±0.58 | 10.00±1.00 | 4.33±0.58 | 4.00±1.00 | 3.00±0.63 |
| 9- Kolibri | Apical | 3.00±0.00 | 7.67±1.15 | **9.00±0.00** | 9.00±0.00 | 4.00±0.00 | 3.00±0.00 | 3.00±0.00 | 1.83±0.41 |
|  | Central | 5.33±0.58 | 9.33±0.58 | **10.00±0.00** | 10.00±0.00 | 5.00±0.00 | 4.00±0.00 | 4.00±0.00 | 3.00±0.00 |
|  | Basal | 4.33±1.53 | 9.33±0.58 | **9.67±0.58** | 9.33±0.58 | 5.00±0.00 | 4.00±0.00 | 4.00±0.00 | 2.33±0.82 |
| 10- Ralle | Apical | 4.67±1.53 | 8.33±0.58 | **9.00±0.00** | 9.33±0.58 | 4.00±0.00 | 3.33±0.58 | 3.00±0.00 | 1.80±0.45 |
|  | Central | 5.67±1.53 | 9.67±0.58 | **10.00±0.00** | 10.00±0.00 | 4.33±0.58 | 4.33±0.58 | 3.67±0.58 | 2.40±0.55 |
|  | Basal | 6.00±1.73 | 9.67±0.58 | **10.00±0.00** | 9.67±0.58 | 4.33±0.58 | 4.00±0.00 | 3.00±0.00 | 2.60±0.55 |
| 11- Nandu | Apical | 5.00±0.00 | 9.50±0.71 | **9.67±0.58** | 10.00±0.00 | 9.67±0.58 | 9.33±0.58 | 3.33±0.58 | 1.83±0.41 |
|  | Central | 5.67±0.58 | 10.00±0.00 | **11.00±0.00** | 11.00±0.00 | 10.33±0.58 | 10.00±0.00 | 4.00±0.00 | 3.00±0.00 |
|  | Basal | 6.33±1.53 | 10.50±0.71 | **10.33±0.58** | 10.33±0.58 | 11.33±0.58 | 11.33±0.58 | 4.00±0.00 | 2.50±0.84 |
| 12- Fasan | Apical | 4.00±1.00 | 8.00±1.41 | **9.00±0.00** | 9.00±1.00 | 8.67±0.58 | 4.00±0.00 | 3.00±0.00 | 1.50±0.84 |
|  | Central | 5.67±1.53 | 9.50±0.71 | **10.00±0.00** | 10.33±0.58 | 9.33±0.58 | 4.00±0.00 | 4.00±0.00 | 2.83±0.41 |
|  | Basal | 6.00±1.73 | 9.50±0.71 | **10.67±0.58** | 11.00±0.00 | 10.33±0.58 | 4.00±0.00 | 3.67±0.58 | 2.67±1.37 |

Data are presented as the mean [±](http://www.baidu.com/link?url=duy-wQqaYmgEkMTManTS8fcdGS-xbMeKDPsL9DSBR6AwBaLBFd1-kWBE4IDWU3glXrNYbHrT81vdNQf8u8feiq) SD, n=6 for PM stage, and n=3 for the remaining stages. The bold text suggests maximum floret primordia number stage is GA stage, and the grey boxes indicate the time windows of floral degradation occrued.

**Table S8.** Living floret (primordia) number per spikelet in apical, central and basal spikelets at seven floral developmental stages and grain number per spikelet at physiological maturity (PM) in twelve detillered genotypes (tiller removal) grown in the greenhouse.

| **Cultivars (detillering, greenhouse)** | **Position** | **TS** | **WA** | **GA** | **YA** | **TP** | **HD** | **AN** | **PM** |
| --- | --- | --- | --- | --- | --- | --- | --- | --- | --- |
| 1- Adlung’s Alemannen | Apical | 3.33±0.58 | 9.00±0.00 | **10.00±0.00** | 10.00±0.00 | 10.00±0.00 | 4.00±0.00 | 3.67±0.58 | 0.17±0.41 |
|  | Central | 4.33±0.58 | 9.33±0.58 | **11.00±0.00** | 11.00±0.00 | 10.00±0.00 | 5.00±0.00 | 4.67±0.58 | 4.33±0.82 |
|  | Basal | 4.67±0.58 | 9.00±0.00 | **11.00±0.00** | 11.00±0.00 | 10.67±0.58 | 5.00±1.00 | 5.00±1.00 | 4.67±0.52 |
| 2- NOS Nordgau | Apical | 4.00±1.00 | 9.00±0.00 | **9.67±0.58** | 9.33±0.58 | 9.33±0.58 | 3.67±0.58 | 3.00±0.00 | 0.40±0.89 |
|  | Central | 5.00±1.00 | 9.50±0.71 | **10.67±0.58** | 11.00±0.00 | 11.00±0.00 | 4.67±0.58 | 4.33±0.58 | 3.60±0.55 |
|  | Basal | 5.00±1.00 | 10.00±1.41 | **10.33±0.58** | 10.33±0.58 | 11.33±0.58 | 5.00±0.00 | 4.67±0.58 | 3.80±0.84 |
| 3- Peragis Garant | Apical | 5.00±2.00 | 8.33±0.00 | **9.67±1.15** | 10.00±0.00 | 10.00±0.00 | 10.00±0.00 | 3.67±0.58 | 1.83±1.60 |
|  | Central | 6.67±1.53 | 9.00±1.15 | **10.67±1.15** | 11.67±0.58 | 11.00±0.00 | 11.33±0.58 | 5.33±0.58 | 2.83±1.72 |
|  | Basal | 7.33±1.15 | 9.67±0.58 | **10.67±0.00** | 10.67±0.58 | 11.00±0.00 | 11.00±0.00 | 5.33±1.15 | 3.50±0.84 |
| 4- Heine’s Peko | Apical | 2.00±0.00 | 8.50±0.71 | **9.67±0.58** | 10.00±0.00 | 9.67±0.58 | 3.67±0.58 | 3.33±0.58 | 0.00±0.00 |
|  | Central | 3.67±1.15 | 10.00±0.00 | **10.33±0.58** | 10.33±0.58 | 11.33±0.58 | 5.00±0.00 | 5.00±0.00 | 1.50±1.64 |
|  | Basal | 3.00±1.00 | 10.50±0.71 | **11.00±0.00** | 12.00±1.00 | 11.00±1.00 | 5.00±0.00 | 4.67±0.58 | 3.83±0.41 |
| 5- Hohenheimer Franken II | Apical | 2.33±0.58 | 7.67±0.58 | **10.00±0.00** | 9.67±0.58 | 5.67±2.89 | 4.00±0.00 | 3.33±0.58 | 0.00±0.00 |
|  | Central | 3.33±0.58 | 8.67±0.58 | **10.00±0.00** | 10.00±0.00 | 8.67±2.31 | 5.33±0.58 | 4.00±0.00 | 1.17±1.33 |
|  | Basal | 3.00±1.00 | 9.33±0.58 | **9.67±0.58** | 10.33±0.58 | 8.00±1.73 | 5.00±1.00 | 4.00±0.00 | 3.33±1.21 |
| 6- Probat | Apical | 2.00±0.00 | 8.33±0.58 | **10.33±0.58** | 11.00±0.00 | 10.67±0.58 | 8.33±3.79 | 4.00±1.00 | 0.00±0.00 |
|  | Central | 3.00±0.00 | 10.33±0.58 | **11.00±1.00** | 11.67±0.58 | 12.33±0.58 | 10.00±3.61 | 5.33±0.58 | 2.00±1.63 |
|  | Basal | 3.33±1.15 | 10.33±0.58 | **11.33±0.58** | 12.00±1.00 | 12.00±1.00 | 10.33±4.62 | 5.67±0.58 | 3.25±0.50 |
| 7- Breustedt’s Lera | Apical | 3.00±0.00 | 8.33±0.58 | **9.33±0.58** | 9.67±0.58 | 9.33±0.58 | 4.00±0.00 | 3.00±0.00 | 0.00±0.00 |
|  | Central | 5.33±0.58 | 9.33±0.58 | **10.33±0.58** | 10.67±0.58 | 12.00±0.00 | 6.00±0.00 | 5.00±0.00 | 2.40±2.07 |
|  | Basal | 5.00±0.00 | 9.33±0.58 | **11.00±0.00** | 11.00±1.00 | 11.67±0.58 | 6.00±0.00 | 5.00±0.00 | 3.20±1.79 |
| 8- Arin | Apical | 3.67±0.58 | 7.33±0.58 | **8.67±0.58** | 9.67±0.58 | 9.33±0.58 | 4.33±0.58 | 3.00±0.00 | 2.17±0.75 |
|  | Central | 5.67±1.15 | 8.00±0.00 | **10.33±1.15** | 10.00±1.00 | 10.67±0.58 | 5.33±0.58 | 4.67±0.58 | 4.50±0.55 |
|  | Basal | 7.00±1.00 | 9.67±0.58 | **10.67±0.58** | 11.00±0.00 | 10.67±0.58 | 5.33±0.58 | 4.67±0.58 | 4.00±1.10 |
| 9- Kolibri | Apical | 4.00±1.00 | 8.33±0.58 | **9.50±0.71** | 10.00±0.00 | 4.00±0.00 | 4.00±0.00 | 4.00±0.00 | 2.83±0.41 |
|  | Central | 4.67±0.58 | 8.67±0.58 | **10.00±0.00** | 10.33±0.58 | 5.00±0.00 | 5.00±0.00 | 5.00±0.00 | 4.17±0.41 |
|  | Basal | 4.00±1.00 | 8.33±0.58 | **10.00±0.00** | 10.00±0.00 | 5.00±0.00 | 5.00±0.00 | 4.67±0.58 | 4.33±0.52 |
| 10- Ralle | Apical | 5.00±0.00 | 8.67±0.58 | **9.33±0.58** | 9.00±0.00 | 9.67±1.15 | 4.33±0.58 | 4.00±0.00 | 1.50±0.84 |
|  | Central | 5.67±0.58 | 10.00±0.00 | **10.33±0.58** | 11.00±0.00 | 10.00±0.00 | 5.00±0.00 | 4.67±0.58 | 3.50±0.55 |
|  | Basal | 6.00±1.00 | 9.67±0.58 | **10.67±0.58** | 10.33±0.58 | 10.67±0.58 | 5.00±0.00 | 4.33±0.58 | 3.50±0.55 |
| 11- Nandu | Apical | 4.33±1.15 | 9.67±0.58 | **10.00±0.00** | 9.67±1.15 | 10.33±0.58 | 10.67±0.58 | 4.00±0.00 | 1.83±0.98 |
|  | Central | 5.33±0.58 | 10.33±1.15 | **11.67±0.58** | 11.33±0.58 | 11.67±0.58 | 11.33±1.53 | 5.33±0.58 | 4.50±0.55 |
|  | Basal | 4.67±0.58 | 10.67±0.58 | **11.33±0.58** | 11.33±0.58 | 11.67±0.58 | 12.00±0.00 | 5.33±0.58 | 4.17±0.98 |
| 12- Fasan | Apical | 2.00±0.00 | 8.00±0.00 | **9.00±0.00** | 9.67±0.58 | 10.00±0.00 | 10.00±0.00 | 3.00±0.00 | 1.17±0.75 |
|  | Central | 4.00±0.00 | 9.33±0.58 | **10.67±0.58** | 11.00±0.00 | 10.50±0.71 | 10.67±0.58 | 4.33±0.58 | 3.83±0.98 |
|  | Basal | 3.67±0.58 | 10.00±0.00 | **11.33±1.15** | 11.00±1.00 | 10.00±0.00 | 11.00±0.00 | 4.67±0.58 | 3.50±0.55 |

Data are presented as the mean [±](http://www.baidu.com/link?url=duy-wQqaYmgEkMTManTS8fcdGS-xbMeKDPsL9DSBR6AwBaLBFd1-kWBE4IDWU3glXrNYbHrT81vdNQf8u8feiq) SD, n=6 for PM stage, and n=3 for the remaining stages. The bold text suggests maximum floret primordia number stage is GA stage, and the grey boxes indicate the time windows of floral degradation occrued.

**Table S9.** Thermal time required for seven floral developmental stages in twelve spring wheat cultivars grown in the field (control and tiller removal).

| Cultivars | TS | | WA | | GA | | YA | | TP | | HD | | AN | |
| --- | --- | --- | --- | --- | --- | --- | --- | --- | --- | --- | --- | --- | --- | --- |
|  | Control | Detilering | Control | Detilering | Control | Detilering | Control | Detilering | Control | Detilering | Control | Detilering | Control | Detilering |
| 1- Adlung’s Alemannen | 761±0a | 761±0a | 1032±34a | 1032±0a | 1094±0a | 1094±0a | 1125±0b | 1162±0a | 1182±17a | 1207±0a | 1243±0a | 1266±21a | 1314±0b | 1410±0a |
| 2- NOS Nordgau | 761±0a | 761±0a | 1012±0a | 1032±0a | 1094±0a | 1094±0a | 1162±0a | 1162±0a | 1172±17b | 1207±0a | 1207±0a | 1231±41a | 1314±0a | 1314±0a |
| 3- Peragis Garant | 761±0a | 739±0a | 973±0a | 973±0a | 1032±0a | 1032±0a | 1080±0a | 1094±0a | 1125±21a | 1137±0a | 1192±0a | 1182±0a | 1314±0a | 1314±0a |
| 4- Heine’s Peko | 837±0a | 837±0a | 1032±0a | 10730a | 1094±0b | 1162±0a | 1162±0b | 1207±0a | 1219±0b | 1243±21a | 1290±0a | 1314±17a | 1352±0a | 1366±0a |
| 5- Hohenheimer Franken II | 837±0a | 837±0a | 1032±0a | 1080±0a | 1094±0b | 1125±0a | 1172±0a | 1192±0a | 1231±0b | 1314±0a | 1267±0b | 1352±0a | 1335±0b | 1410±0a |
| 6- Probat | 837±0a | 824±0a | 1032±0a | 1032±0a | 1080±0b | 1162±0a | 1146±0b | 1184±0a | 1231±21b | 1366±0a | 1314±0b | 1410±0a | 1366±0a | 1443±0a |
| 7- Breustedt’s Lera | 837±0a | 824±0a | 1080±0a | 1105±0a | 1125±0b | 1192±0a | 1192±0b | 1243±0a | 1243±21b | 1314±18a | 1290±0b | 1366±17a | 1352±0b | 1388±0a |
| 8- Arin | 761±0a | 739±0a | 973±0a | 973±0a | 1032±0b | 1080±0a | 1080±0b | 1125±0a | 1125±0a | 1144±0a | 1162±0a | 1162±0a | 1266±0b | 1314±0a |
| 9- Kolibri | 761±0a | 761±0a | 973±0a | 973±0a | 1032±0b | 1089±0a | 1080±0a | 1094±0a | 1137±28a | 1105±0a | 1182±0a | 1172±0a | 1290±0a | 1314±0a |
| 10- Ralle | 761±0a | 739±0a | 973±0a | 973±0a | 1032±0b | 1094±0a | 1080±0b | 1125±0a | 1125±21b | 1162±0a | 1192±0a | 1207±0a | 1314±0a | 1331±0a |
| 11- Nandu | 761±0a | 739±0a | 973±0a | 955±0a | 1032±0a | 1048±0a | 1080±0a | 1080±0a | 1146±21a | 1125±0a | 1162±0a | 1162±0a | 1267±0a | 1267±0a |
| 12- Fasan | 761±0a | 753±0a | 973±0a | 973±0a | 1085±0a | 1080±0a | 1094±0a | 1125±0a | 1137±0a | 1162±26a | 1172±0b | 1207±0a | 1314±0a | 1314±0a |
| Average | 786±36a | 776±40a | 1005±37a | 1014±50a | 1069±33b | 1104±47a | 1121±43b | 1149±48a | 1173±47a | 1207±79a | 1222±54a | 1252±81a | 1316±32b | 1349±51a |

Data are presented as the mean[±](http://www.baidu.com/link?url=duy-wQqaYmgEkMTManTS8fcdGS-xbMeKDPsL9DSBR6AwBaLBFd1-kWBE4IDWU3glXrNYbHrT81vdNQf8u8feiq)SD, n=3; different letters per trait indicate significant differences between control and treated plants (*p*<0.05).

**Table S10.** Thermal time required for seven floral developmental stages in twelve spring wheat cultivars grown in the greenhouse (control and tiller removal).

| Cultivars | TS | | WA | | GA | | YA | | TP | | HD | | AN | |
| --- | --- | --- | --- | --- | --- | --- | --- | --- | --- | --- | --- | --- | --- | --- |
|  | Control | Detilering | Control | Detilering | Control | Detilering | Control | Detilering | Control | Detilering | Control | Detilering | Control | Detilering |
| 1- Adlung’s Alemannen | 785±0a | 785±0a | 947±0b | 983±0a | 1037±73b | 1091±0a | 1073±10b | 1163±0a | 1145±64b | 1217±10a | 1145±0b | 1271±0a | 1253±0b | 1433±21a |
| 2- NOS Nordgau | 785±0a | 785±0a | 983±0a | 911±0b | 1067±0a | 1073±0a | 1097±0a | 1127±0a | 1208±0a | 1151±0b | 1163±0b | 1199±0a | 1253±0a | 1277±62a |
| 3- Peragis Garant | 749±0a | 731±0a | 911±0a | 899±0a | 947±0a | 947±0a | 1001±0a | 983±0a | 1055±0b | 1085±21a | 1109±0b | 1145±36a | 1217±0a | 1217±0a |
| 4- Heine’s Peko | 785±0a | 785±0a | 983±0a | 1073±42a | 1091±0b | 1163±0a | 1181±0b | 1217±0a | 1217±0b | 1277±21a | 1253±0b | 1289±0a | 1289±0b | 1469±0a |
| 5- Hohenheimer Franken II | 785±0a | 785±0a | 1025±42a | 1073±0a | 1163±0a | 1163±0a | 1217±0a | 1229±42a | 1265±21a | 1277±21a | 1313±0a | 1301±21a | 1397±0b | 1469±25a |
| 6- Probat | 821±0a | 749±0a | 1001±0a | 1007±21a | 1109±0a | 1127±0a | 1217±0a | 1163±b | 1241±21a | 1205±10a | 1271±0a | 1229±21b | 1301±21b | 1349±21a |
| 7- Breustedt’s Lera | 857±0a | 857±0a | 1067±0a | 1163±0a | 1163±0b | 1217±0a | 1217±0b | 1265±0a | 1277±10b | 1337±0a | 1307±0b | 1373±0a | 1397±0b | 1487±0a |
| 8- Arin | 749±0a | 749±0a | 923±0a | 935±21a | 1073±0a | 1037±0b | 1145±0a | 1073±0a | 1181±21a | 1145±0b | 1217±0a | 1181±0b | 1289±0a | 1253±0a |
| 9- Kolibri | 749±0a | 731±0a | 911±0a | 911±21a | 1001±42a | 983±0a | 1055±0a | 1019±0b | 1079±0a | 1073±0a | 1145±0a | 1145±0a | 1217±21a | 1217±21a |
| 10- Ralle | 713±0a | 731±0a | 947±10a | 923±0a | 1019±0a | 1019±0a | 1073±0a | 1073±0a | 1133±21a | 1145±21a | 1163±42a | 1181±21a | 1253±0b | 1289±0a |
| 11- Nandu | 749±0a | 749±0a | 947±0a | 971±0a | 1043±0a | 1073±0a | 1091±0a | 1109±0a | 1145±21a | 1145±0a | 1199±25a | 1217±0a | 1277±21a | 1265±21a |
| 12- Fasan | 785±0a | 773±0a | 983±21a | 983±0a | 1019±0b | 1061±0a | 1103±0b | 1163±0a | 1229±0a | 1199±0a | 1235±0a | 1235±0a | 1277±0b | 1313±0a |
| Average | 776±37a | 768±35a | 969±48a | 986±80a | 1061±66a | 1080±76a | 1123±70a | 1132±83a | 1181±73a | 1188±79a | 1210±67a | 1231±67a | 1285±58b | 1337±99a |

Data are presented as the mean[±](http://www.baidu.com/link?url=duy-wQqaYmgEkMTManTS8fcdGS-xbMeKDPsL9DSBR6AwBaLBFd1-kWBE4IDWU3glXrNYbHrT81vdNQf8u8feiq)SD, n=3; different letters per trait indicate significant differences between control and treated plants (*p*<0.05).

**Table S11.** Spikelet fertility (%) in twelve genotypes under control and tiller removal treatments in the greenhouse and field at harvest.

| Genotype | Year of release | Greenhouse | | Field | |
| --- | --- | --- | --- | --- | --- |
|  |  | Control | Detillering | Control | Detillering |
| 1- Adlung’s Alemannen | 1931 | 99.21±1.94a | 62.65±8.27b | 95.78±4.95a | 80.73±12.63b |
| 2- NOS Nordgau | 1933 | 87.12±4.74a | 70.69±8.07b | 92.80±5.57a | 96.01±3.73a |
| 3- Peragis Garant | 1946 | 84.89±11.32a | 69.24±22.60a | 95.03±3.17a | 96.39±6.70a |
| 4- Heine’s Peko | 1947 | 63.80±8.89a | 48.83±9.77b | 96.41±5.61a | 88.25±9.57b |
| 5- Hohenheimer Franken II | 1951 | 77.91±16.77a | 49.35±3.11b | 90.15±13.47a | 71.68±11.50b |
| 6- Probat | 1953 | 65.73±11.74a | 50.97±12.93a | 97.33±3.27a | 83.49±5.35b |
| 7- Breustedt’s Lera | 1959 | 62.44±11.44a | 48.69±12.67a | 94.13±4.52a | 84.06±9.05b |
| 8- Arin | 1962 | 67.17±5.11b | 86.21±8.94a | 99.07±2.27a | 95.74±5.90a |
| 9- Kolibri | 1966 | 88.39±9.39a | 90.93±6.66a | 95.95±5.83a | 97.07±5.28a |
| 10- Ralle | 1980 | 92.11±4.69a | 88.04±9.96a | 92.73±5.31a | 78.03±13.26b |
| 11- Nandu | 1988 | 83.71±13.98a | 85.92±15.70a | 92.19±5.96a | 96.63±3.50a |
| 12- Fasan | 1997 | 71.66±12.43a | 73.49±3.29a | 97.83±3.64a | 83.81±14.32b |

Data are presented as the mean[±](http://www.baidu.com/link?url=duy-wQqaYmgEkMTManTS8fcdGS-xbMeKDPsL9DSBR6AwBaLBFd1-kWBE4IDWU3glXrNYbHrT81vdNQf8u8feiq)SD, n=6; different letters per trait indicate significant differences between control and treated plants (*p*<0.05).


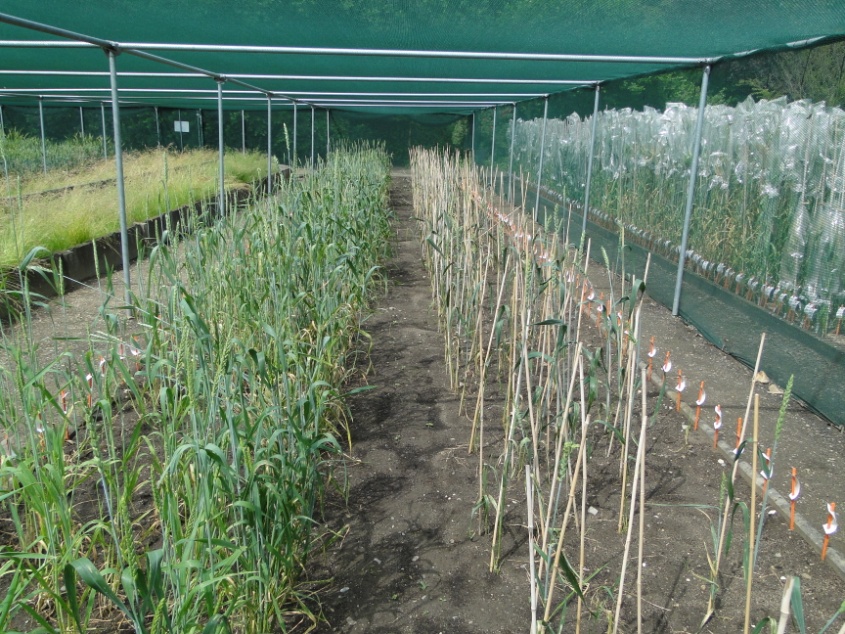

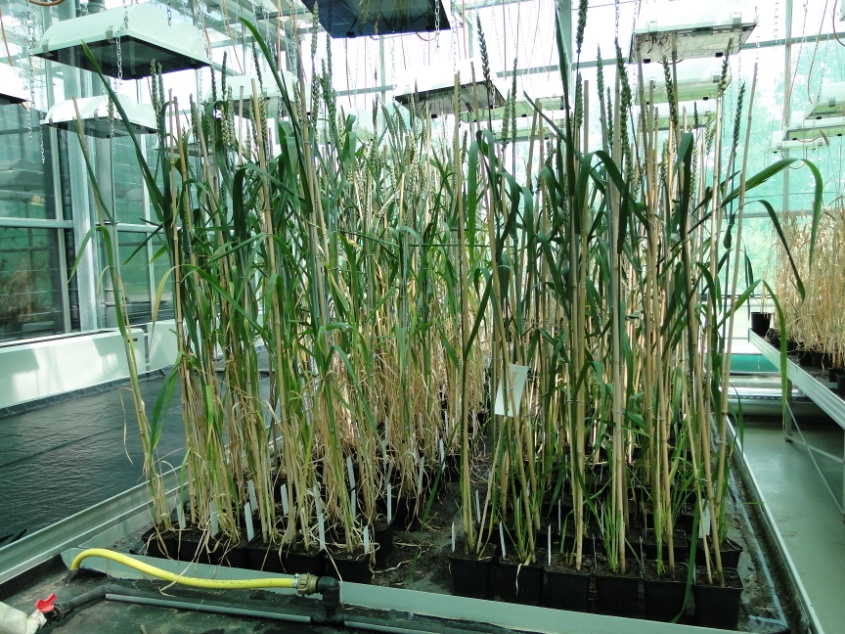


**b**

**a**

**Fig. S1.** Control and tiller removal experiments in field (a) and greenhouse (b).

**Fig. S2.** Maximum floret primordia number per spike at apical, central and basal spikelets in twelve genotypes (1-12) and averages of genotypes (13) under control and detillering treatments in field (mean [±](http://www.baidu.com/link?url=duy-wQqaYmgEkMTManTS8fcdGS-xbMeKDPsL9DSBR6AwBaLBFd1-kWBE4IDWU3glXrNYbHrT81vdNQf8u8feiq) SD, n=3).

**C**

**Fig. S3.** Fertile floret number per spike at apical, central and basal spikelets in twelve genotypes (1-12) and averages of genotypes (13) under control and detillering treatments in field (mean [±](http://www.baidu.com/link?url=duy-wQqaYmgEkMTManTS8fcdGS-xbMeKDPsL9DSBR6AwBaLBFd1-kWBE4IDWU3glXrNYbHrT81vdNQf8u8feiq) SD, n=3).

**Fig. S4.** Final grain number per spike at apical, central and basal spikelets in twelve genotypes (1-12) and averages of genotypes (13) under control and detillering treatments in field (mean [±](http://www.baidu.com/link?url=duy-wQqaYmgEkMTManTS8fcdGS-xbMeKDPsL9DSBR6AwBaLBFd1-kWBE4IDWU3glXrNYbHrT81vdNQf8u8feiq) SD, n=6).

**Fig. S5.** Boxplots of control and tiller removal leaf area (cm^2^), leaf dry weight (g), spike dry weight (g), main stem dry weight (g) at the GA stage and significant levels of difference between control and tiller removal. * p< 0.05; **p < 0.01. Each boxplot displays the data of the twelve genotypes for corresponding traits at GA stage; the significances suggest comprehensive influence of detillering on different traits of the twelve genotypes.

**Fig. S6.** Boxplots of main stem dry weight (g) (main shoot), spike chaff (g) (main shoot) at PM stage and significant levels of difference between control and tiller removal. **p< 0.01; ***p< 0.001. Each boxplot displays the data of the twelve genotypes for corresponding traits at PM stage, the significances suggest comprehensive influence of detillering on different traits of the twelve genotypes.
